# Bio-Based Flexible Solar-Driven Sustainable Generator with Efficient Electricity Generation Enabled by Plant Transpiration System

**DOI:** 10.1007/s40820-025-01960-5

**Published:** 2026-01-13

**Authors:** Lingli Kong, Junjie Lu, Tianwen Luo, Bai Huang, Lihua Fu, Baofeng Lin, Chuanhui Xu

**Affiliations:** University Engineering Research Center of Green Chemical New Materials, School of Chemistry and Chemical Engineering, Guangxi University, Nanning, 530004 Guangxi People’s Republic of China

**Keywords:** Photothermal self-healing, Ionic conductivity, Sustainable generation, Elastomer

## Abstract

The global energy crisis and electricity shortage pose unprecedented challenges. Bio-based solar-driven ionic power generation devices with flexibility, photothermal self-healing and scalability hold great promise for sustainable electricity and alleviating energy crisis. Here, inspired by plant transpiration, a multifunctional bio-based ion conductive elastomer with solar power generation capability was designed by engineered synergy among epoxy natural rubber, cellulose nanofibrils, lithium bis(trifluoromethane) sulfonimide and eumelanin. The film exhibits an outstanding stretchability (1072%) and toughness (22.7 MJ m^−3^). The favorable synergy of low thermal conductivity, high hygroscopicity and photothermal conversion performance endowed the film with a large thermal gradient under light illumination, driving efficient water transpiration. Furthermore, the excellent interfacial compatibility between eumelanin and matrix facilitates the formation of space charge regions, which further enhances Li^+^ transport. The film demonstrates excellent evaporation rate (2.83 kg m^−2^ h^−1^), output voltage (0.47 V) and conductivity (5.11 × 10^–2^ S m^−1^). Notably, the film exhibits remarkable photothermal self-healing performance even in saline environment, achieving 99.6% healing efficiency of output voltage. Therefore, the film demonstrates significant prospects for applications in photo-thermoelectric generation and solar-driven ionic power generation.

**Supplementary Information:**

The online version contains supplementary material available at 10.1007/s40820-025-01960-5.

## Highlights

- Bio-based solar-driven ionic power generation devices were designed based on the principle of plant transpiration, achieving an evaporation rate of 2.83 kg m⁻^2^ h⁻^1^ and an output voltage of 0.47 V.
- The excellent interfacial compatibility between eumelanin and the matrix facilitates the formation of space charge layer, which significantly enhances Li^+^ transport.
- Solar-driven ionic power generation devices possess excellent photothermal self-healing ability (99.6% healing efficiency) and stretchability (1072%) in saline environment.

**Supplementary Information:**

The online version contains supplementary material available at 10.1007/s40820-025-01960-5.

## Graphical abstract

**Figure:**
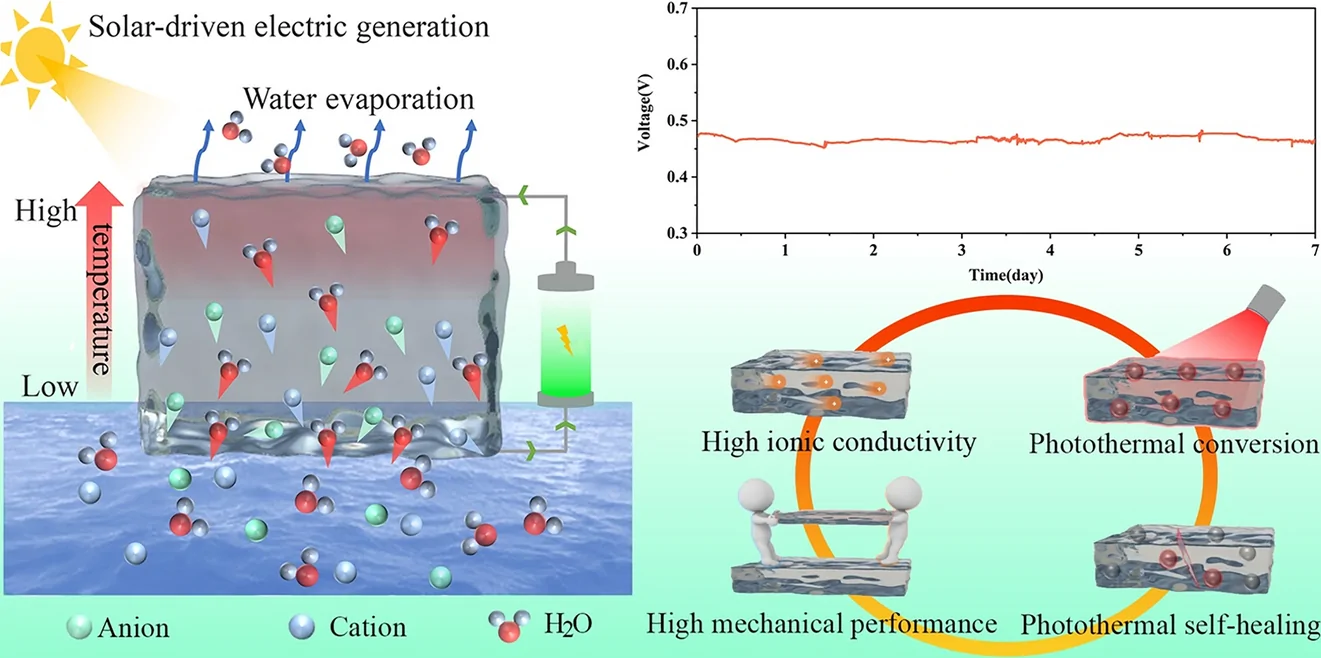

## Introduction

With the environmental deterioration and exhaustion of fossil energy sources, replacing fossil-based energy with green energy generated from renewable resources has become imperative [1–4]. To address these challenges, it is crucial to develop integrated solutions for sustainable development. Recently, technologies for hydrovoltaic power generation, especially moisture-enabled electricity generation (MEG) [5, 6], have garnered great attention due to their spontaneous direct current output, but have their own limitations arising from unsustainable electricity output [7]. Solar energy, as an inexhaustible and eco-friendly energy source, has attracted considerable attention in the production of green electricity [8–10]. In fact, most of the solar energy absorbed by the Earth’s surface is converted into heat, and approximately half of this thermal energy was used to natural water evaporation [11]. Therefore, based on the Soret and electrokinetic effects [12], the development of an integrated system that can utilize abundant solar thermal resources to achieve sustainable power generation during the process of driving natural water evaporation, would provide ideas for alleviating the global energy crisis and developing green electricity.

In recent years, the integration of solar evaporators and power generation demonstrates the potential for solar-driven water evaporation technology to enable sustainable electricity production [13]. For example, Mu’s group [14] prepared a thermoelectricity-fresh-water cogenerator via using a thermoelectric generator (TEG) and a starch-polyacrylamide (S-PAM) hydrogel. The evaporation rate and voltage output of cogenerator can achieve 1.79 kg m^−2^ h^−1^ and 0.09 V, respectively. Zhou et al. [15] prepared an integrated system by carbon nanotubes (CNTs) modified filter paper and Nafion membrane, and the system possesses an output voltage of 0.062 V and evaporation rate of 1.15 kg m^−2^ h^−1^. Xu et al. [16] designed a hybrid fabric composed of basalt fibers and cotton yarns with asymmetric structure by textile weaving technology, followed by depositing carbon black on the top layer through flame combustion to facilitate photothermal conversion. The fabric exhibits output voltage of 0.064 V and evaporation rate of 1.52 kg m^−2^ h^−1^. Wang’s group [17] realized effective solar-thermal-electro integration based on reduced graphene oxide (RGO)/Mn_3_O_4_/Al_2_O_3_ composite nanomaterials, with evaporation rates and output voltages of 1.74 kg m^−2^ h^−1^ and 0.352 V, respectively. Obviously, although they have achieved solar-driven water evaporation for power generation via careful design, the evaporation rate is still relatively low, which greatly affects the electric output. In addition, their integrated systems are typically assembled from separate modules with complex and inflexible designs, which unfortunately not only damage the efficiency of solar evaporation and power generation, but also lacks flexibility to meet the requirements of complex application conditions. More importantly, due to torsion, tearing, friction and compression in practical applications, solar power generation devices are inevitably susceptible to mechanical damage that significantly reduces the service life of devices and impaired functional reliability. Therefore, the development of excellent evaporation rate, high output voltage, remarkable flexibility and photothermal self-healing bio-based solar-driven ionic power generation device is crucial for extending device lifespan and alleviating energy crises.

In general, the excellent photothermal conversion materials play a vital role in highly solar-driven evaporation. Currently, most of the reported photothermal conversion materials in the field of solar power generation are mainly graphene [18], CNTs [19–21], MXene [22–24], semiconductors [25, 26] and metal nanoparticles [3, 27]. Although these materials exhibit excellent solar absorption properties, their fabrication is complicated and costly. Compared to these, the eumelanin extracted from the ink sac of cuttlefish seems to have more advantages in the field of solar power generation. Most of the time, the ink sacs of cuttlefish are discarded after slaughter. Around the world, the amount of discarded ink sacs would be huge. Therefore, as the main component of ink sacs, the sources of eumelanin are more abundant, cheap and sustainable [28]. More importantly, eumelanin has excellent photothermal conversion ability and biocompatibility [29, 30]. For example, Yang et al. [31] fabricated a light-controlled healable and reversible adhesive elastomers via dispersing eumelanin uniformly in carboxylic styrene butadiene rubber (XSBR). The photothermal conversion efficiency of elastomers can reach 80%. Jin et al. [32] prepared a phytic acid–decorated melanin/κ-carrageenan aerogels (PMCAs) using the cationic induction method, whose photothermal conversion efficiency is as high as 89%. Therefore, the eumelanin can be one of the ideal photothermal materials for fabricating solar-driven ionic power generation devices.

**Fig. 1:**
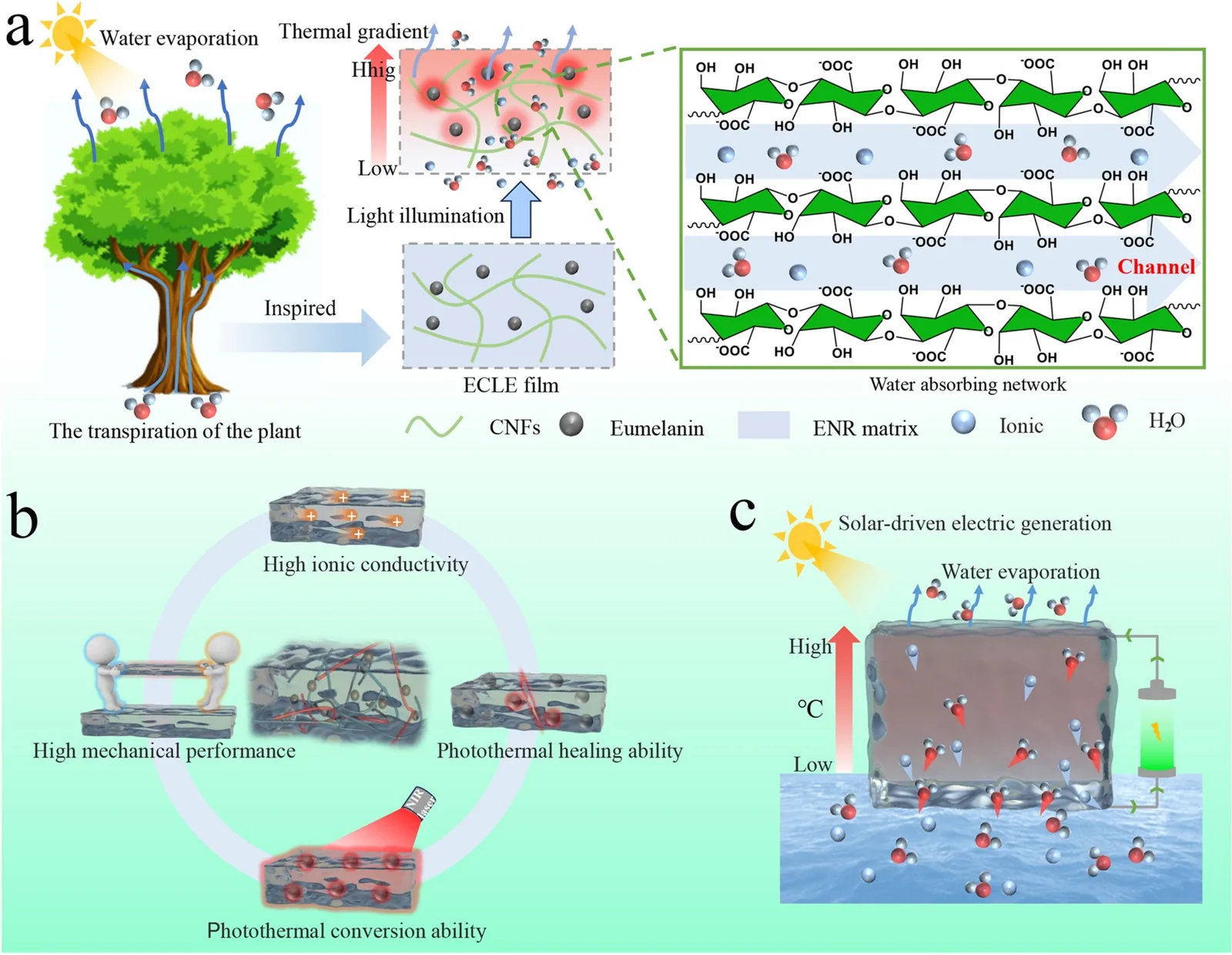
Conceptual design, performance and application. **a** Schematic illustration of the design of ECLE with a “Plant Transpiration”-like system. **b** Function of ECLE. **c** Schematic illustration of solar-driven ionic power generation

Transpiration of plants primarily consists of three stages [33]: water absorption by the roots, water transport through the stems and water evaporation from the leaves. Various ions and water are absorbed by the roots of plants and transported to the leaves, and then the water was released into the air in the form of water vapor. Inspired by this phenomenon, we propose eco-friendly and convenient latex film-forming method to fabricate an epoxy natural rubber (ENR)/cellulose nanofibrils (CNFs)/lithium bis(trifluoromethane) sulfonimide (LiTFSI)/eumelanin bio-based elastomer (termed ECLE) with excellent evaporation rate, high conductivity, remarkable flexibility and photothermal self-healing for solar-driven ionic power generation and photo-thermoelectric generation. ENR serves as the flexible matrix in ECLE, and then LiTFSI, eumelanin and CNFs were dispersed in the matrix through non-covalent interactions. CNFs with hygroscopicity and abundant polar groups construct a water-absorbing network in the matrix, providing channels for water evaporation and ion transport, while eumelanin with strong light absorption provides energy through photothermal conversion for water evaporation, ion transport and photothermal self-healing (Fig. 1a). Notably, the excellent interfacial compatibility between eumelanin and the matrix facilitates the formation of space charge layer, which significantly enhances Li^+^ transport. The engineered synergy endows ECLE with a “Plant Transpiration”-like system and superior multifunctional properties, including outstanding photothermal conversion ability, high stretchability, efficient photothermal self-healing and high ionic conductivity (Fig. 1b). Benefiting from the favorable synergy of low thermal conductivity, high hygroscopicity and photothermal conversion performance, the ECLE possess excellent evaporation rate and output voltage. Surprisingly, the ECLE retains remarkable photothermal self-healing performance even in saline environment. The ECLE also exhibit scalability in the solar-driven ionic power generation process. Specifically, the output voltage of ECLE can be adjusted by the number of ECLE units. By simply connecting ECLE units in series, the ECLE devices can provide sufficient power for many common small electronic devices without any additional auxiliary devices. This work opens new possibilities for the development of efficient evaporation rate, flexibility, photothermal self-healing and scalable sustainable green power generation systems (Fig. 1c).

## Experimental Section

### Materials

Epoxidized natural rubber (ENR) latex with an epoxidation degree of 40% was provided by the Agricultural Products Processing Research Institute of Chinese Academy of Tropical Agricultural Science (Zhanjiang, China). Cellulose nanofibrils (CNFs, TEMPO oxidized) was purchased from Science Co., Ltd. Bis(trifluoromethane) sulfonimide lithium salt (LiTFSI, 99.9%) was obtained from Aladdin Co., Ltd. SiO_2_ nanoparticles with an average particle size of about 150–200 nm was supplied by Hebei Yigui Welding Materials Co., Ltd.

### Preparation of Eumelanin, ECLE and ECLS

#### Preparation of Eumelanin

Eumelanin was extracted from ink sacs of cuttlefish which were purchased from a local seafood market (Nanning, China). Eumelanin was dispersed in deionized water and centrifuged (1000 rpm) for 10 min to remove larger particles, followed by sonicated for 30 min using an ultrasonic bath to separate the eumelanin. A dispersed solution of eumelanin was obtained after several washing, centrifugation. Finally, the dispersed solution of eumelanin was lyophilized (− 40 °C, ~ 50 Pa) to obtain a solid eumelanin powder.

#### Preparation of ENR/CNFs/LiTFSI/Eumelanin (ECLE) Films

First, the solid eumelanin powder was dispersed in deionized water and sonicated for 60 min using an ultrasonic bath to obtain the dispersed solution of eumelanin. CNFs and LiTFSI was together dissolved in deionized water, respectively. Then, under mechanical stirring of 600 rpm, the dispersed solution of eumelanin, CNFs solution and LiTFSI solution were slowly dropped into ENR latex (17.0 g, containing 5 g of pure ENR), respectively. After 1 h, the latex was removed bubbles using a planetary mixing/degassing apparatus. Ultimately, the films were obtained by drying at 40 °C. The films were named ECLE-x, where x represents the content of eumelanin.

#### Preparation of ENR/CNFs/LiTFSI/SiO2 (ECLS) Films

The SiO_2_ (1 phr) powder was dispersed in CNFs (3 phr) solution and sonicated for 60 min using an ultrasonic bath to obtain the mixed solution of SiO_2_/CNFs. LiTFSI (4 phr) was dissolved in deionized water. Then, under mechanical stirring of 600 rpm, the mixed solution of SiO_2_/CNFs and LiTFSI were slowly dropped into ENR latex (17.0 g, containing 5 g of pure ENR), respectively. After 1 h, the latex was removed bubbles using a planetary mixing/degassing apparatus. Ultimately, the ECLS films were obtained by drying at 40 °C. The ECLS films were named ECLS-1.

### Characterizations

Fourier transform infrared (FTIR) spectra were recorded in attenuated total reflection model from 4000 to 400 cm^−1^ with resolution of 4 cm^−1^ (Nexus-470, Thermo Fisher Scientific, USA). Differential scanning calorimetry (DSC) analysis was performed using a METTLER DSC (Mettler Toledo, Swiss) tester. The testing was taken from − 80 to 160 °C with rate of 5 °C min^−1^ in nitrogen atmosphere. The thermogravimetric analysis (TGA) was investigated by a TGA/DSC (Mettler Toledo, stare system) with rate of 10 K min^−1^ (30 to 800 °C) under nitrogen atmosphere. The EDS and SEM were recorded by a scanning electron microcope (SEM, Hitachi S3400 N, Japan) at 5 kV. Prior to test, the samples were coated on a thin layer of gold. Transmission electron microscopy (TEM) was carried out to observe the structure of the samples using JEM1400PLUS under accelerating voltage of 300 kV. The XPS was carried out using amonochromatic Al Kax X-ray source on X-ray photoelectron spectroscopy (XPS, Thermo Kalpha). The X-ray diffraction (XRD) was conducted using a D/max-Ultima IV X-ray diffractometer with Cu Kα radiation (λ = 1.5418 Å). The experimental parameters included a current of 30 mA, a voltage of 40 kV and an angular range of 5° to 50° scanned at a rate of 10° min^−1^ with steps of 0.02°. The UV–Vis-NIR spectra were measured using a UV1901 spectrometer (Yoke Instrument, China) in the wavelength range from 1100 to 200 nm with a resolution of 0.5 nm. The potential of different parts of the sample were conducted using Kelvin probe force microscopy (KPFM) (Bruker). The water contact angle (WCA) was performed at room temperature using DSA100E (KRÜSS, Germany). The size of the water droplet employed in the test was 4 μL. Stress relaxation experiment was carried out on a TA Q850. The samples were balanced at the set temperature for 5 min, and then a 5% strain was applied on the samples, testing for 60 min.

## Results and Discussion

### Structural Characteristics and Mechanical Properties of ECLE

**Fig. 2:**
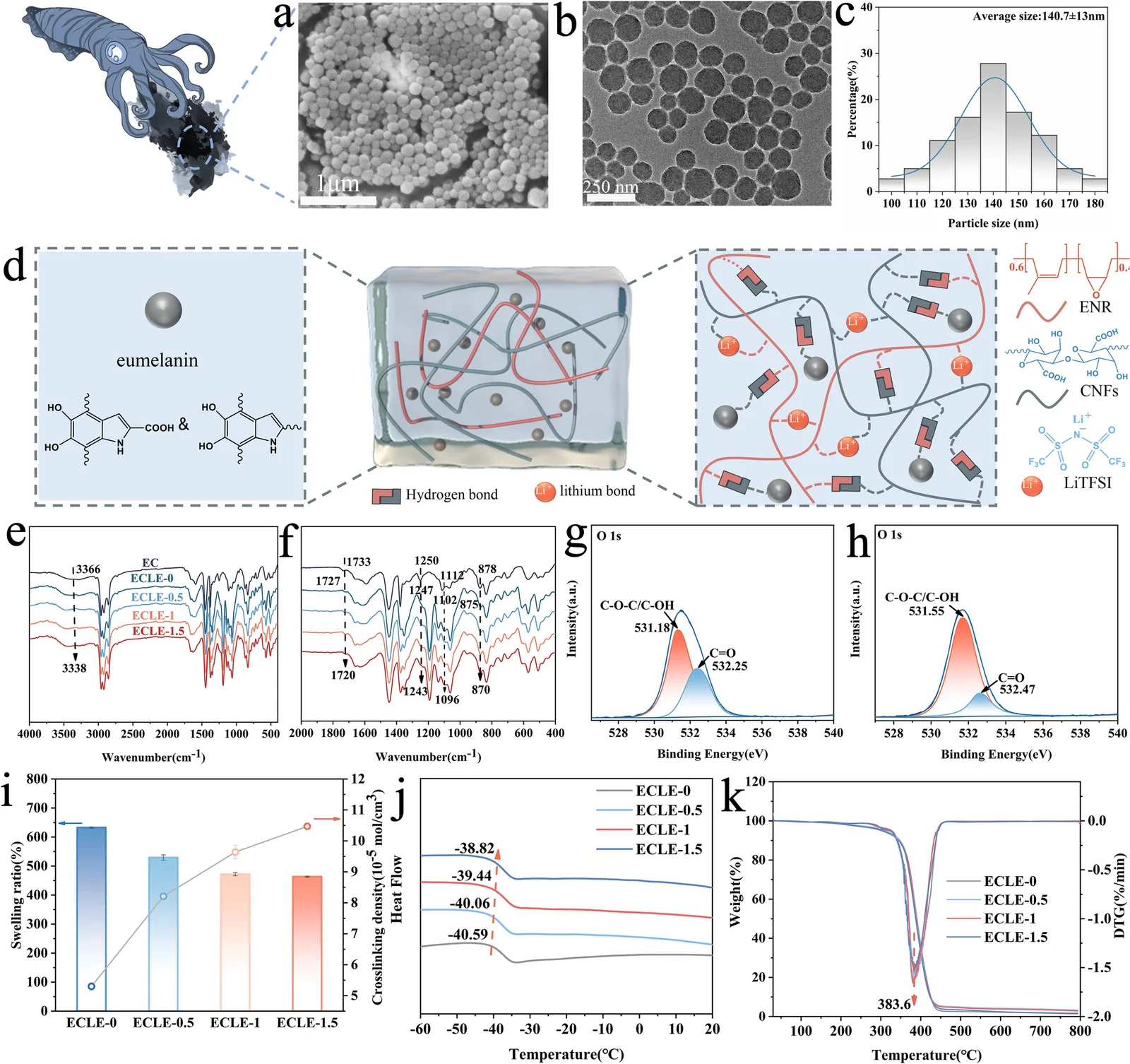
Characterization of eumelanin and ECLE. **a** SEM and **b** TEM images of eumelanin. **c** Size distribution of eumelanin. **d** Structural schematic illustration of ECLE. **e, f** FTIR spectra of ECLE with different E content. **g** XPS spectra of ECLE-0. **h** XPS spectra of ECLE-1. **i** Cross-linking densities and swelling of ECLE. **j** DSC curves of ECLE. **k** TG curves of ECLE

Inspired by plant transpiration, ECLE with a “Plant Transpiration”-like system was fabricated. The eumelanin with an average diameter of 140.7 ± 13 nm was extracted from the ink sac of cuttlefish (Fig. 2a–c), and it serves as a solar absorber with excellent photothermal conversion efficiency, equivalent to the leaves of plants. A large number of hydrogen and lithium bonds were formed between ENR, CNFs, eumelanin and LiTFSI to construct a stable and elastic network during film formation (Fig. 2d). Then, the CNFs with excellent hygroscopicity form a water-absorbing network within the ECLE that facilitates water/ion transport, mimicking the roles of plant roots and stems. The ENR matrix provides the necessary flexibility for practical applications. The structure of the ECLE network was verified using FTIR. As shown in Fig. 2e, f, the absorption peaks at 3366, 1733 and 1112 cm^−1^ were attributed to the stretching vibrations of –OH, C = O and C–O–C of CNFs, respectively [34]. The vibrational peaks at 1250 and 878 cm^−1^ were assigned to the epoxy group from ENR. Compared to EC, upon the introduction of LiTFSI, the C = O and C–O–C of CNFs and epoxy group of ENR have undergone red-shift to 1727, 1102, 1247 and 875 cm^−1^, respectively. This indicates that ionic bonds have been formed between Li^+^ and oxygen atoms [35]. Meanwhile, in comparison with the ECLE-0, the absorption peaks of ECLE with different eumelanin content underwent an obvious red-shift upon the further addition of eumelanin, indicating the formation of hydrogen bonds between CNFs, ENR and eumelanin. Besides, the internal interactions of ECLM film are further analyzed by XPS. As shown in Fig. 2g and h, the 0 1*s* spectra of ECLE-0 were fitted at 531.18 and 532.25 eV, corresponding to two characteristic peaks of –OH/C–O–C and C = O, respectively [36]. After adding eumelanin, the peaks of –OH/C–O–C and C = O of ECLE-1 shifted to higher binding energies (531.55 and 532.47 eV, respectively). This further illustrates the formation of hydrogen bonds.

Such a physically crosslinked network was confirmed via an equilibrium swelling experiment. The ECLE could be easily picked up by a nipper and remained their complete shapes after being soaked in toluene for 4 days (Fig. S1). As shown in Fig. 2i, with the increase in eumelanin content, the cross-linking density of ECLE increased from 5.3 × 10^−5^ mol cm^−3^ of ECLE-0 to 10.5 × 10^−5^ mol cm^−3^ of ECLE-1.5, and correspondingly the swelling ratio decreased from 632% of ECLE-0 to 463% of the ECLE-1.5 due to that the formation of physical cross-linking in ECLE enforced a restriction on the molecular chain movement of ENR [37, 38]. The DSC results further supported this conclusion, with the glass transition temperature (T_g_) increasing from − 40.59 °C of ECLE-0 to − 38.82 °C of ECLE-1.5 (Fig. 2j). However, the T_g_ value was still much lower than the room temperature, and the ENR maintained enough chain mobility over a wide temperature range to achieve self-healing and high ionic conductivity for the ECLE. Figure 2k shows the thermal stability of ECLE with different eumelanin content. The thermal decomposition temperatures of material exceed 380 °C, which suggested that the ECLE possess excellent thermal stability for photothermal conversion applications operating below 200 °C.

**Fig. 3:**
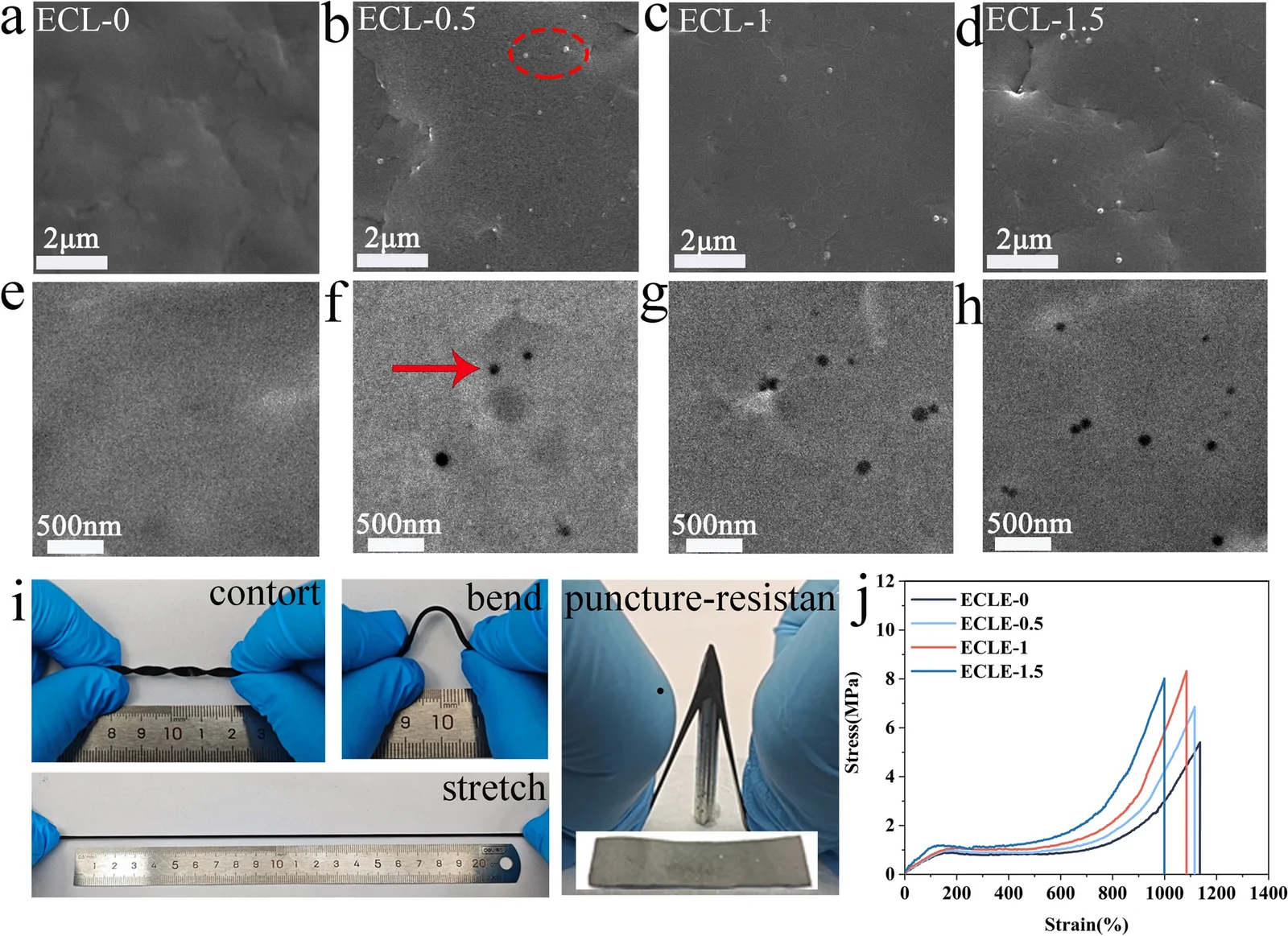
Microstructure and mechanical properties. **a-d** SEM of ECLE. **e–h** TEM of ECLE. **i** Photographs showing the ECLE being stretched, contorted, bended and puncture-resistant. **j** Stress–strain curves of ECLE

The dispersibility of eumelanin and LiTFSI was critical to the “transpiration” operation of the ECLE. The characteristic elements (S and F) of LiTFSI were uniformly distributed, suggesting that the LiTFSI is homogeneously dispersion in the ECLE (Fig. S2). As shown in Fig. 3a-h, various concentrations of eumelanin demonstrate uniform dispersion and excellent interfacial compatibility in the ENR. According to molecular dynamics simulation (Fig. S3), the binding energy between eumelanin and CNFs in the ECLE was calculated to be − 10.28 kcal mol^−1^, which was higher than that between eumelanin and ENR (− 0.54 kcal mol^−1^), demonstrating that the eumelanin preferentially forms hydrogen bonds with CNFs to achieve uniform dispersion [39]. Benefiting from the uniform dispersion of eumelanin and the formation of crosslinked networks, the ECLE demonstrates superior mechanical strength. As shown in Fig. 3i, ECLE can be contorted, bended and stretched without rupture and withstand puncture under large deformation. Figure 3j shows that the stress–strain curve exhibits obvious strain hardening behavior, which is attributed to the occurrence of strain-induced crystallization (Fig. S4). Therefore, when the concentration of eumelanin reaches 1.5 phr, the tensile stress, Young’s modulus and toughness of ECLE-1.5 can be achieved 8.28 MPa, 3.16 MPa and 23.13 MJ m^−3^, respectively, while the elongations at break exceeds 950% (Fig. S5). The presence of such remarkable mechanical properties is highly necessary in the field of solar-driven ionic electric generation.

### Photothermal Conversion and Photothermal Self-healing Properties of ECLE

**Fig. 4:**
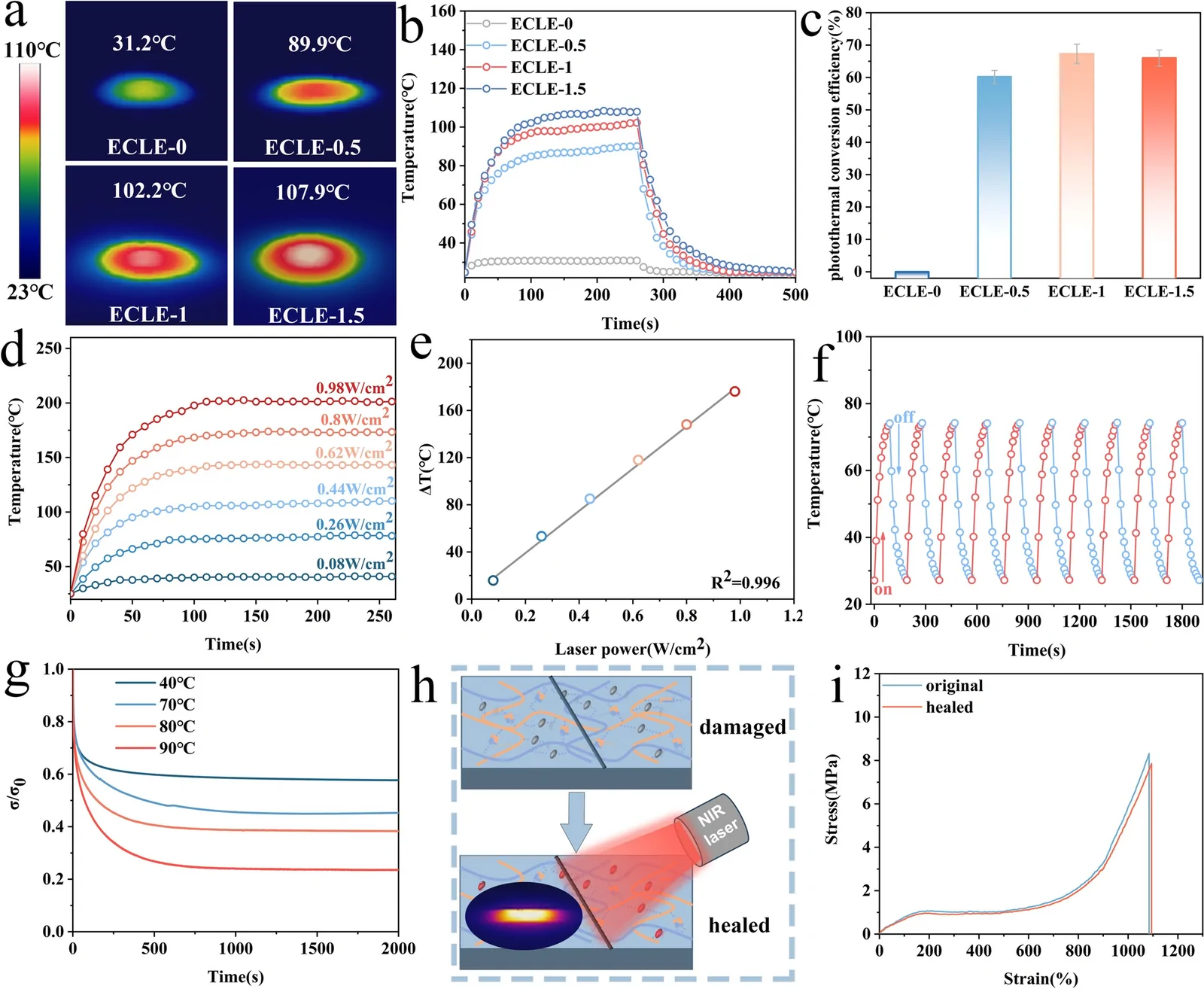
Photothermal conversion performance and photothermal self-healing. **a** NIR digital photo of ECLE with different eumelanin content.** b** Surface temperature–time curves of ECLE with different eumelanin content. **c** Photothermal conversion efficiency of ECLE. **d** Surface temperature–time curves of ECLE at different laser power. **e** Linear fitting of temperature and laser power for ECLE-1. **f** Cyclic photothermal conversion performance of ECLE-1 under 0.26 W cm^−2^. **g** Stress relaxation curves of ECLE-1 at different temperature. **h** Schematic diagram of photothermal self-healing. **i** Stress–strain curves of ECLE-1 after photothermal self-healing at 0.26 W cm^−2^ for 2 h

The ECLE demonstrates excellent light absorption capacity (Fig. S6a). Compared with ECLE-0, the ECLE-1.5 can absorb ~ 99% light (200–1100 nm). The ultrahigh light absorption capacity provides feasibility for excellent photothermal conversion performance. To evaluate the photothermal conversion performance of ECLE, the near-infrared (NIR) laser with a wavelength of 808 nm was used to irradiate samples, and the surface temperature variation of samples over time was recorded with an infrared thermal imager (Fig. S7a). Figure 4a and b showcases the NIR photo and temperature–time curves of ECLE at NIR laser irradiation of 0.44 W cm^−2^. The surface temperature of ECLE-0 only increased to 31.2 °C owing to the lack of eumelanin. After the introduction of eumelanin, the surface temperature of ECLE increases from 89.9 °C of ECLE-0.5 to 107.9 °C of ECLE-1.5. This is because eumelanin particles absorb light energy, causing electrons inside the particles to move from lower energy states to higher energy states. Then, the heat is generated by enhancing the internal vibrations of molecules through non-radiative jumps [40]. The photothermal conversion efficiency (η*) of ECLE was further calculated (for details, please see the Supporting Information). As depicts in Figs. 4c and S8, the η* value of ECLE-1 reaches 67.3%, which is beyond most of the photothermal materials. The temperature change of ECLE-1 was evaluated at different laser power to realize the temperature tuning (Fig. 4d). When a laser power was applied to the ECLE-1, its temperature increases sharply within 60 s. As shown, the temperature of ECLE-1 increased from ~ 41.1 to ~ 200.2 °C as the laser power rose from 0.08 to 0.98 W cm^−2^, demonstrating a strong linear fitting relationship with laser power (Fig. 4e). Such a good linear fitting relationship suggests that the temperature of ECLE films can be precisely tuned by varying laser power. In addition, the ECLE-1 retains excellent photothermal conversion stability under switching NIR laser irradiation for multiple cycles (Fig. 4f). Even at high NIR laser power, ECLE-1 still exhibits similar results (Fig. S7b). The above results demonstrate that the ECLE have stable photothermal conversion performance, providing feasibility for the transpiration effect and showing promising potential in photo-thermoelectric conversion, photothermal self-healing and solar-driven ionic power generation.

The ECLE-1 exhibits typical stress relaxation at different temperatures (Fig. 4g). Within the same time (500 s), the σ/σ_0_ value decreased from 0.59 at 40 °C to 0.26 at 90 °C. Evidently, stress relaxation behavior verifies the reversibility of non-covalent crosslink network, which can effectively release stress via rearrangement of lithium and hydrogen bonds [41]. The reversible characteristics of non-covalent interactions and photothermal conversion performance endow ECLE with photothermal self-healing ability. Therefore, the self-healing behavior of ECLE-1 was evaluated at 0.26 W cm^−2^ and the schematic diagram of NIR light-controlled healing is shown in Fig. 4h. After 2 h of NIR exposure, the ECLE-1 can withstand large strain and the weight of 1 kg without breaking (Fig. S9a). The internal and surface cutting marks almost disappeared after self-healing (Fig. S9b, c). Figure 3i reveals that the ECLE-1 restored 7.86 MPa of its original stress and achieved 95% healing efficiency. The excellent photothermal self-healing capability is attributed to the uniform dispersion of eumelanin in the ECLE, which effectively transfers the heat energy that from photothermal conversion uniformly within the matrix, promoting the exchange of non-dynamic covalent bonds and diffusion of the ENR molecular chains.

### Ionic Conductivity of ECLE

**Fig. 5:**
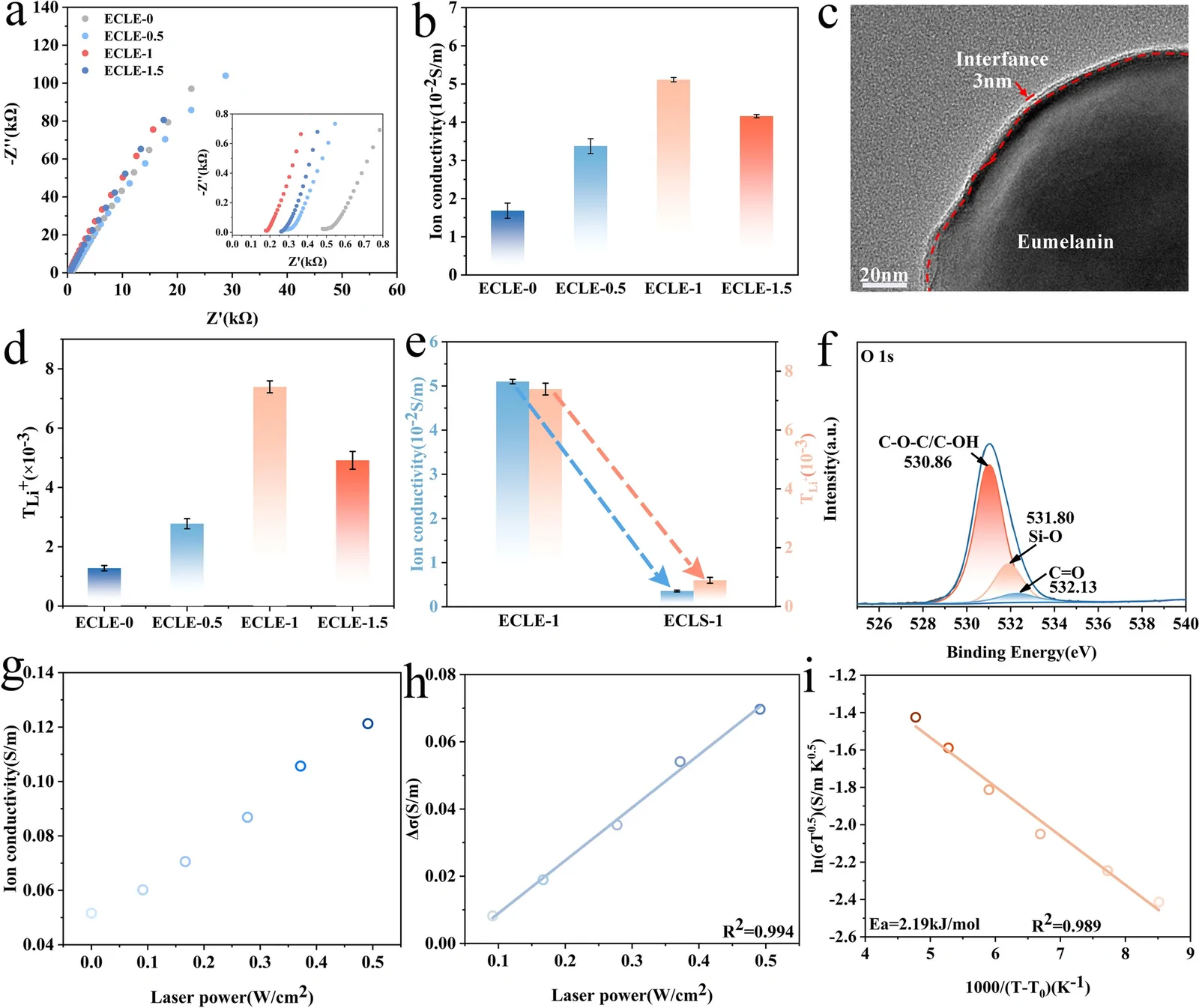
Conductivity of ECLE.** a** Nyquist plot of ECLE. **b** Ionic conductivity of ECLE. **c** TEM images of the ECLE-1. **d** Ionic transference number of ECLE. **e** Ionic transference number and ionic conductivity of ECLS-1. **f** XPS spectra of ECLS-1. **g** Ionic conductivity of ECLE-1 at different laser powers. **h** Ionic conductivity of ECLE-1 versus laser powers. **i** VTF-σ plots for the ionic conductivity of ECLE-1

To achieve efficient solar-driven ionic power generation, the excellent conductivity of ECLE is crucial for charge transfer to harvest the generated electricity [42]. The ion conductivity originates from the transport of lithium ions within the ECLE system [43], which is measured by the electrochemical impedance spectroscopy (EIS). As shown in Fig. 5a, b, the conductivity of ECLE increases from 1.68 × 10^−2^ to 5.11 × 10^−2^ S m^−1^ as the eumelanin content increased from 0 to1 phr. With further increased the eumelanin content to 1.5 phr, the conductivity of ECLE-1.5 decreased to 4.15 × 10^−2^ S m^−1^. This may be due to the excessive eumelanin can forms more cross-linking points, which limits the movement of polymer chains, resulting in a decrease in conductivity. The TEM image (Fig. 5c) confirms an interface layer of ~ 3 nm formed between the ENR matrix and eumelanin, which is attributed to that the abundance of polar groups on eumelanin surface facilitates robust interfacial adhesion to the ENR matrix. Such a continuous eumelanin/ENR interface phase can form a space charge region [44], promoting Li^+^ transport between the two phases [45, 46]. This result is consistent with the report by Li et al. [47]. The ionic transference number (T_Li+_) of ECLE further verifies that the eumelanin promotes Li^+^ transport. As shown in Figs. 5d and S10a-d, the T_Li+_ of ECLE-0 is only 1.3 × 10^−3^. Upon reaching 1 phr eumelanin content, the T_Li+_ of ECLE-1 increased to 7.4 × 10^−3^, which is 5.6 times that of ECLE-0. For comparison, the surface hydrophobic modified nano silica was used to fabricate ion conductive films (ECLS-1), with the same preparation process and formula as ECLE. The results show that the conductivity (3.5 × 10^−3^ S m^−1^) and T_Li+_ (8.8 × 10^−4^) of ECLS-1 are both lower than that those of ECLE-1 (Figs. 5e and S10e). The XPS spectra reveal that the binding energy of ECLS-1 is lower than that of ECLE-1 (Figs. 5f and 2h). The above results indicate that the eumelanin form a space charge region with the ENR to facilitate Li^+^ transport. Furthermore, the influence of photothermal on the conductivity of ECLE has also been investigated. As shown in Fig. 5g, the conductivity of ECLE-1 increases from 0.051 to 0.121 S m^−1^, as the laser power rises from 0 to 0.49 W cm^−2^. Laser power exhibits a strong linear fitting relationship with Δσ of ECLE-1 (Fig. 5h), showing an excellent precise controllability of conductivity of ECLE. The enhanced conductivity is attributed to photothermal effect, where the temperature rise enhances motion of molecular chains and facilitates the ion transport [48]. Figure S11 presents the Arrhenius plots of the ECLE-1, which illustrates that the ionic conductivities increased regularly with temperature without sudden sharp increases. Moreover, the ECLE-1 exhibits a low activation energy (Ea) of only 2.19 kJ mol⁻^1^ (Fig. 5i), indicating its significant potential for rapid ion conduction [49]. The synergistic integration of high conductivity and excellent transpiration effect is expected to achieve outstanding output voltage in solar-driven ionic power generation process.

### Solar Power Generation Performance of ECLE

**Fig. 6:**
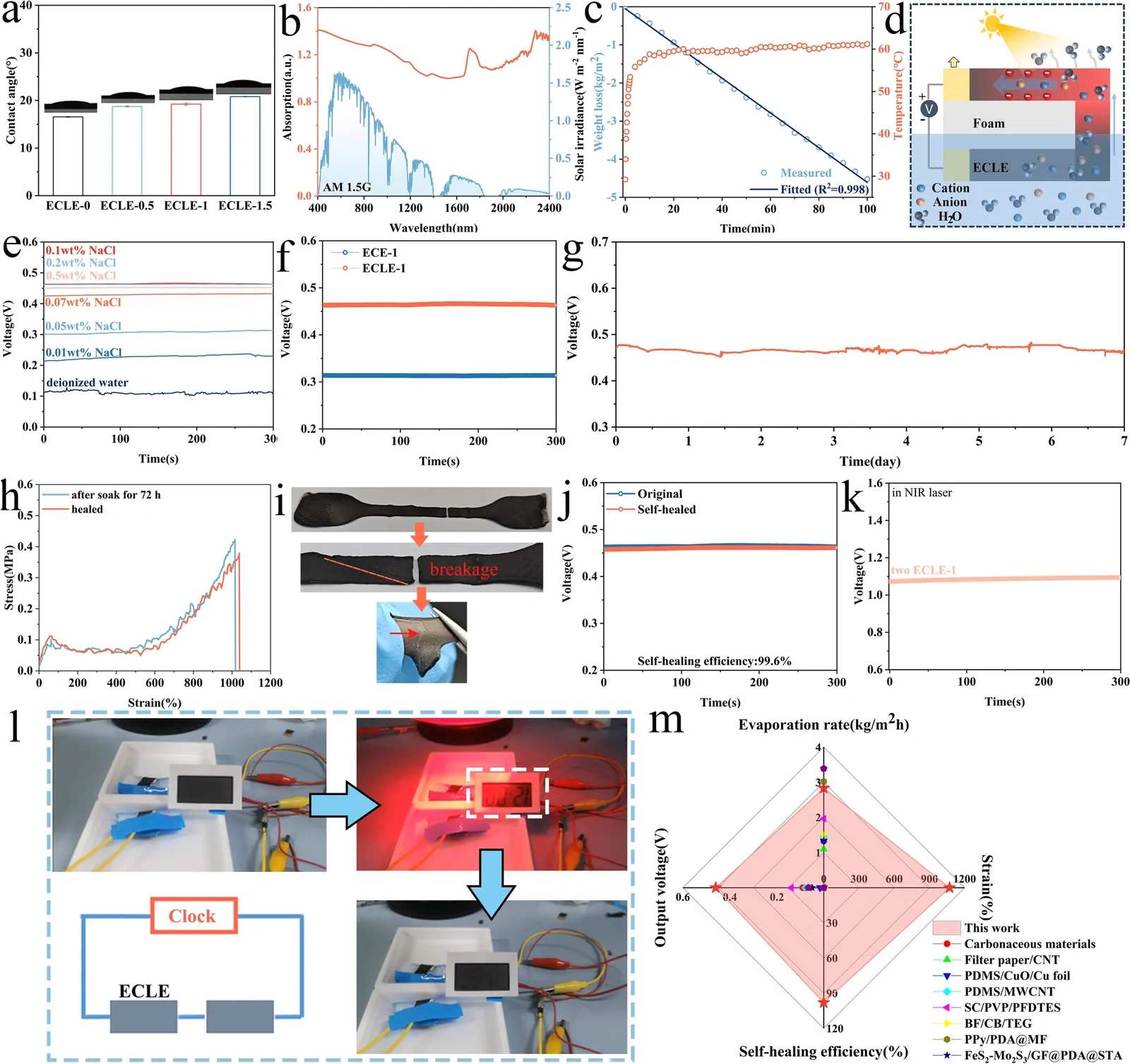
Solar-driven ionic power generation and self-healing.** a** Water contact angle of ECLE surface. **b** UV–Vis-NIR spectra for the ECLE-1 and the solar spectrum (AM 1.5G). **c** Time-dependent surface temperature of the ECLE-1 and the loss of water from the ECLE-1 under 0.15 W cm^−2^ laser irradiation. **d** Schematic of the device. **e** Output voltage of the ECLE-1 at different NaCl concentration under the NIR laser irradiation of 0.15 W cm^−2^. **f** The output voltage of the ECLE-1 and ECE-1. **g** Stability of output voltage of the ECLE-1 under 0.15 W cm^−2^. NIR laser irradiation. **h** Stress–strain curve of ECLE-1 soaked in 0.1 wt% NaCl solution for 72 h and the healed sample. **i** Photo of healed ECLE-1 being break. **j** Output voltage of the ECLE-1 and healed ECLE-1. **k** Output voltage of the two ECLE-1 devices. **l** Photo of the clock being lit. **m** Comparison of ECLE with previously reported solar-driven steam power generation devices in terms of strain, output voltage, water evaporation rate and self-healing ability [15, 16, 19, 27, 51–55]

The excellent hydrophilicity and strong light absorption are essential for the efficient transpiration of ECLEs (Fig. 6a, b). As shown in Fig. 6c, the surface temperature of the ECLE-1 quickly rises to ~ 61 °C within 10 min under a NIR laser irradiation of 0.15 W cm^−2^. The infrared image shows a significant thermal gradient (~ 21.5 °C) at the interface between the ECLE-1 and the water (Fig. S12a). This high thermal gradient can be attributed to the low thermal conductivity of ECLE-1 (0.184 W m^−1^ K^−1^), which is significantly lower than that of other materials used for solar photothermal conversion such as metal nanoparticles, organic polymers and carbons organic polymers (0.4–100 W m^−1^ K^−1^) [11]. As a result, the ECLE-1 achieves water evaporation rate as high as 2.83 kg m^−2^ h^−1^. Based on the work in close collaboration between light absorption and low thermal conductivity to promote efficient interfacial solar evaporation, a transpiration effect like that of plants was achieved for the ECLE-1.

Then, a solar-driven ionic power generation device was designed based on the transpiration effect of ECLE-1 (Fig. 6d). The entire device was floated on an NaCl aqueous (0.1 wt%), and half of the film was immersed in the solution, and then the output voltage of ECLE-1 was measured during water evaporation. An ion concentration gradient was formed between the upper and bottom of the device during water evaporation, which generates a potential. As shown in Fig. S12b, c, ECLE-1 turned out an output voltage as high as 0.51 V with a laser power of 0.3 W cm^−2^. To optimize the output performance, we analyzed the influence of different NaCl concentrations on the output performance of ECLE-1. As shown in Fig. 6e, compared to deionized water (0.1 V), the output voltage of ECLE-1 increased from 0.22 to 0.47 V, corresponding to an increase in NaCl concentration from 0.01 wt% to 0.1 wt%. However, further increasing the concentration of NaCl solution results in an excess of free ions in the flow, which can hinder electron transfer owing to the screen effect [50]. As shown, the output voltage of ECLE-1 begins to decrease once the concentration of NaCl exceeds 0.1 wt%. The impact of cation species and film width on the output property of ECLE-1 were also explored. Compared to the CaCl_2_ (0.37 V) and the FeCl_3_ (0.33 V), the NaCl solution generates a higher voltage (0.47 V) at the same concentration (Fig. S12d). As the ECLE-1 width increased from 1 to 3 cm, the output voltage rises correspondingly from 0.47 to 0.55 V (Fig. S12e). A larger width provides more channels for water and ion transport, generating a larger potential difference. Interestingly, the width of ECLE-1 and output voltage possesses strictly linear relationship (Fig. S12f). In addition to the above factors, the influence of Li^+^ on the output voltage of ECLE-1 has also been studied that the output voltage of ECE-1 without Li^+^ is significantly lower than that of ECLE-1 (Fig. 6f). This indicates that Li^+^ endows ECLE-1 with excellent conductivity, improving its output voltage.

From the standpoint of practical application, the ability of stable operation is a key indicator of device reliability. As shown in Fig. 6g, the long-term stability of output voltage even after 7 days. Furthermore, the characteristic absorption peaks of the FTIR test of ECLE-1 was no significant change after 7 days of testing, indicating that ECLE-1 has good structural stability (Fig. S12g). ECLE-1 also exhibited good reproducibility in the light cyclic experiments (Fig. S12h). Considering the potential application in aquatic environments, the mechanical and photothermal self-healing properties of ECLE-1 were evaluated under solution conditions. Despite immersed in 0.1 wt% NaCl solution for 72 h, the soaked ECLE-1 remains maintained a strain value of 1050%, nearly identical to the non-soaked ECLE-1 (1059%), demonstrating an excellent flexibility (Fig. 6h). Figure S14 illustrates the photothermal self-healing process of ECLE-1 in NaCl solution. The stress–strain curve of the healed ECLE-1 is great similarity to the original one soaked in 0.1 wt% NaCl solution for 72 h (Fig. 6h). The healed efficiency of stress and strain was 93 and 100%, respectively. Meanwhile, the interior of the healing site has been fully healed (Fig. 6i). As expected, the output voltage of the healed ECLE-1 can be restored to 0.468 V, with a corresponding self-healing efficiency of 99.6% (Figs. 6j and S15). In addition, based on the weak alkalinity of seawater, we conducted ECLE self-healing experiments in an alkaline solution with PH = 8. It is interesting that the damaged ECLE-1 can still recover its output voltage to 0.454 V after self-healing in alkaline solution, with a self-healing efficiency of up to 97% (Fig. S16). These results indicate that ECLE-1 exhibits outstanding photothermal self-healing ability, which is crucial for extending the service life of solar-driven ionic power generation device. As a power supply, enhancing the output performance is essential for its applications. The output voltage was increased by connecting two ECLE-1 in series. As shown in Fig. 6k, the output voltage of the two ECLE-1 devices is as high as 1.09 V under 0.15 W cm^−2^ laser irradiation. Therefore, the clock can be successfully lit by the output voltage of the two ECLE-1 integrated generators without any additional components (Figs. 6l, S17a and Video S1). The power density of the two ECLE-1 devices even reached 817.4 μW m^−2^ (Fig. S17b, c). Compared to previously reported solar-driven steam power generation devices, the ECLE possess better comprehensive performance, particularly the fascinating flexibility and photothermal self-healing ability (Fig. 6m).

**Fig. 7:**
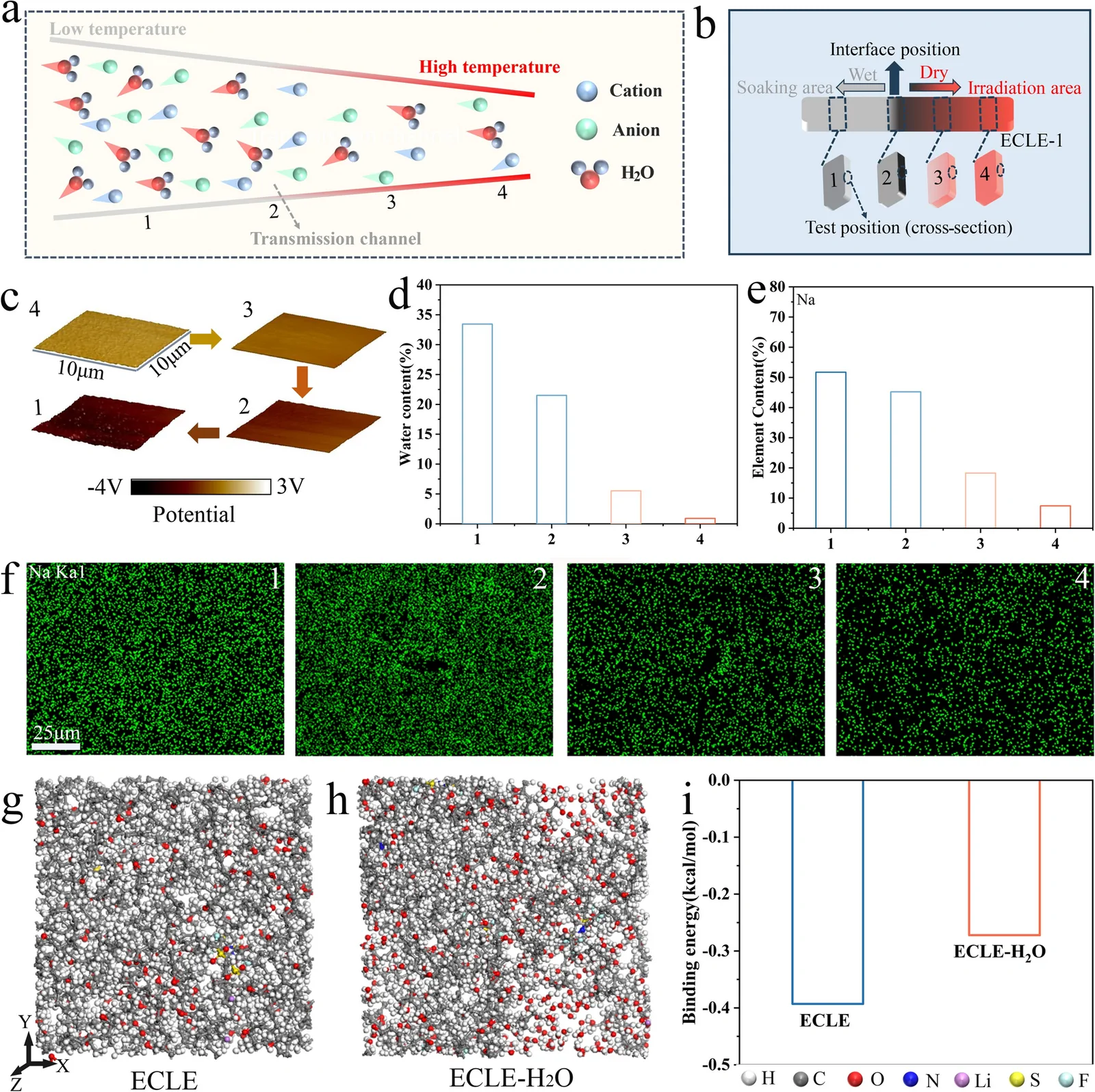
**a** Schematic diagram of water and ion gradient distribution. **b** Schematic diagram of characterization at different locations. **c** KPFM images. **d** Water content at different position. **e** Element content at different position. **f** EDS images at different position. **g** Schematic diagrams of the ECLE structures for molecular dynamics simulation. **h** Schematic diagrams of the ECLE-H_2_O structures for molecular dynamics simulation. **i** Binding energy of ECLE and ECLE-H_2_O systems

So, the ECLE-1 demonstrates outstanding performance in solar-driven ionic power generation based on its distinctive transpiration effect. The CNFs with high hygroscopicity absorb water and provide channels for water transport in solar-driven ionic power generation process. The absorbed water drives directional migrations of ions in the CNFs channels. Then, the uniformly dispersed eumelanin absorbs light and converts it into stable thermal energy, providing consistent heat output for water evaporation and ion transport. According to the Soret effect, the transpiration effect leads to a gradient distribution of ions and water in the ECLE film, generating a potential gradient (Fig. 7a). To confirm this, we selected four cross sections in the ECLE-1 to analyze the distribution of water and ion concentration during solar-driven ionic power generation process. The four cross sections were named 1, 2, 3 and 4 as shown in Fig. 7b. The results in Fig. 7c, d show that the water content decreased from 33.4% of cross-section 1 to 0.9% of cross-section 4. The Kelvin Probe Force Microscopy (KPFM) results also confirmed this result. The varying water content across different positions within the ECLE-1 leads to corresponding differences in their surface potential [56]. Furthermore, the Na^+^ content exhibits a gradient distribution, decreasing from 51.7% of cross-section 1 to 7.4% of cross-section 4 (Figs. 7e, f and S18). Molecular dynamics simulation was used to evaluate the effect of water molecules on the binding energy of structural networks in the ECLE-1. As shown in Fig. 7h and i, the binding energy of ECLE is − 0.393 eV without moisture. After absorbing moisture, the binding energy of ECLE-H_2_O decreases to − 0.272 eV. A lower binding energy facilitates the dissociation and transport of ions within the ECLE [57], thereby improving the electrical signal output of the material.

**Fig. 8:**
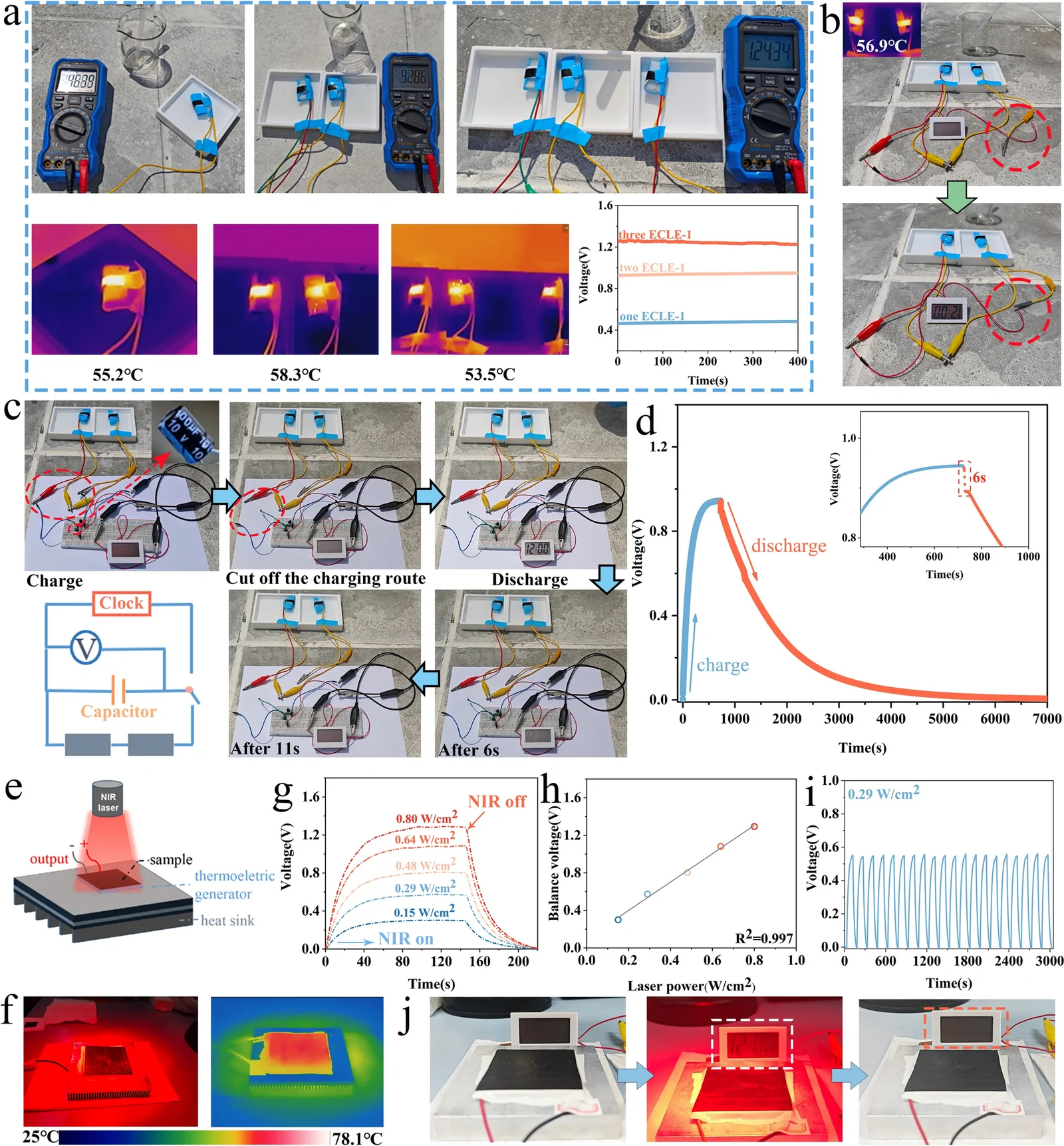
Practical application and thermoelectric generator of ECLE. **a** The output voltage of ECLE-1 (size:1 × 5 cm) device in a natural light environment and NIR images. **b** Photo of two ECLE device lighting up clock. **c** Process of charging and discharging capacitors with two ECLE devices. **d** Voltage variation curve during capacitor charging and discharging. **e** Schematic illustration of the TEG system. **f** Photo of the TEG system and its NIR image. **g** Output voltage of TEG system at different laser power. **h** Relationship between the voltage and power. **i** Cycling voltage generated through multiple heating and cooling cycles. **j** Images of clock work under near-infrared light

To explore the practical feasibility of the solar-driven ionic power generation system, the device was placed in a natural light environment to assess its continuous voltage output. As shown in Figs. 8a and S19, the output voltage of one ECLE-1 device is only 19.6 mV in the absence of sunlight. Remarkably, the ECLE-1 under sunlight shows an output voltage up to 0.48 V, forming a sharp contrast to prior situation. Meanwhile, the surface temperature of ECLE-1 rises to 55.2 °C. The scalability of ECLE in practical applications of power generation has also been explored. The voltage of ECLE can be adjusted by expanding the number of serial units (Fig. 8a). The output voltage reached up to 1.25 V with only three ECLE-1 connection devices. Meanwhile, the clock was successfully lit using two ECLE-1 devices without any additional elements, showing the practical application value of powering devices directly (Fig. 8b and Video S2). Besides, ECLE-1 was used to charge a commercial capacitor without requiring extra rectifiers for energy storage. It only took 10 min to charge capacitors of 100 µF to 0.94 V using two ECLE-1 devices. Subsequently, the charged capacitor can light clock for 6 s during the discharge process (Fig. 8c, d and Video S3). The ECLE-1 devices also exhibit excellent repeatability during charging and discharging processes (Fig. S20). The above results demonstrate the potential of ECLE-1 devices for practical applications in powering devices based on transpiration effect.

In addition to solar-driven ionic power generation, a thermoelectric generator (TEG) system was fabricated by integrating a heat sink, a piece of ECLE-1 and a commercial Seebeck thermoelectric generator (Fig. 8e). Under NIR irradiation, the ECLE-1 rapidly generates heat and transfers it to the upper surface of the thermoelectric generator (Fig. 8f). Based on the Seebeck effect [58], the voltage is generated from thermoelectric generator when a temperature gradient exists between the upper and lower surfaces of the generator. As shown in Fig. 8g, the output voltage of the TEG system increases from 0.3 to 1.3 V as the light power increases from 0.15 to 0.8 W cm^−2^. The output voltage also exhibits a good linear relationship with the laser power, demonstrating that the output voltage of the TEG system can be controlled by varying the light power (Fig. 8h). The TEG system exhibits excellent repeatability through multiple heating and cooling cycles (Fig. 8i). Figure 8j illustrates that the voltage generated by the TEG system can make the clock work (Video S4). Overall, these demonstrations show the potential application of ECLE, including in outdoor portable power devices and energy harvesting systems.

## Conclusions

In summary, we developed a multifunctional bio-based ionic conductive elastomer with a “plant transpiration”-like system for photo-thermoelectric generation and solar-driven ionic power generation. The engineered synergy among ENR, CNFs, LiTFSI and eumelanin endows ECLE with combined advantageous properties simultaneously, including photothermal conversion ability, photothermal self-healing (95% healing efficiency), stretchability (1072%) and toughness (22.7 MJ m^−3^). Based on the combination of molecular chain motion and space charge effect, the conductivity of ECLE is as high as 5.11 × 10^−2^ S m^−1^. Benefiting from the low thermal conductivity, high hygroscopicity and photothermal conversion ability of the ECLE, the excellent transpiration effect can be achieved in solar-driven ionic power generation process. The ECLE exhibits an outstanding evaporation rate (2.83 kg m^−2^ h^−1^) and output voltage (0.47 V). More importantly, the ECLE still exhibits excellent photothermal self-healing in salt solution. The healing efficiency of output voltage can reach 99.6%. Meanwhile, by exploiting the scalability of ECLE, sustainable electricity generation can be achieved in outdoor environments without any other auxiliaries. The ECLE also exhibit excellent output voltage in photo-thermoelectric generation. Therefore, this work offers a promising strategy for the development of efficient, flexibility, photothermal self-healing and scalable sustainable green power generation systems.

## Supplementary Information

Below is the link to the electronic supplementary material.

Supplementary file1 (MP4 3066 KB)

Supplementary file2 (MP4 8236 KB)

Supplementary file3 (MP4 5699 KB)

Supplementary file4 (MP4 2467 KB)

Supplementary file5 (DOCX 5927 KB)
