# Supplementary material for: Bio-Based Flexible Solar-Driven Sustainable Generator with Efficient Electricity Generation Enabled by Plant Transpiration System
Source: Nanomicro Lett. 2026 Jan 13;18:114. doi: 10.1007/s40820-025-01960-5 (PMC12796092; doi:10.1007/s40820-025-01960-5)
Supplement: Supplementary file 5 — Supplementary file5 (DOCX 5927 KB) [file 40820_2025_1960_MOESM5_ESM.docx]

Supporting Information for

**Bio-Based Flexible Solar-Driven Sustainable** **Generator with** **Efficient Electricity Generation Enabled by Plant Transpiration System**

Lingli Kong^1^, Junjie Lu^1^, Tianwen Luo^1^, Bai Huang^1^, Lihua Fu^1^, Baofeng Lin^1^, Chuanhui Xu^1,^*

^1^ University Engineering Research Center of Green Chemical New Materials, School of Chemistry and Chemical Engineering, Guangxi University, Nanning, Guangxi 530004, P. R. China

*Corresponding author. E-mail: [xuhuiyee@gxu.edu.cn](mailto:xuhuiyee@gxu.edu.cn) (Chuanhui Xu)

**S1 Experimental**

**S1.1 Mechanical Performance Test**

Mechanical properties of samples were conducted at room temperature using universal tensiletesting machine (U-CAN UT-2080). The dumbbell-shaped specimens (75 mm × 4 mm × 0.5 mm, ISO 37-2005) were uniaxially stretched to rupture at a strain rate of 500 mm min^-1^ and each sample was teste at three times.

**S1.2 Swelling Ratio and Crosslinking Density of ECLE Test**

The determination of the swelling ratio and crosslinking density of samples was carried out via swelling experiments. The weighed samples (m_0_) were soaked in toluene at room temperature for four days. After that, the samples were carefully extracted from the toluene solution and weighed instantly (m_1_) after removing the toluene from the sample surface. Lastly, the samples were dried at 40°C until they had a constant weight (m_2_). The crosslink density (Ve) can be calculated by equation (S1) [S1]:

$\text{V}_{\text{e}}\text{=-}\frac{\ln\left( \text{1-}\text{V}_{\text{r}} \right)\text{+}\text{V}_{\text{r}}\text{+χ}\text{V}_{\text{r}}^{\text{2}}}{\text{V}_{\text{1}}\text{(}\text{V}_{\text{r}}^{\frac{\text{1}}{\text{3}}}\text{-}\frac{\text{V}_{\text{r}}}{\text{2}}\text{)}}$ (S1)

Among them, V_1_ represents the molar volume of toluene solvent (106.2 cm^3^ mol^-1^) and χ represents the toluene enr interaction parameter (0.34). The volume fraction Vr of rubber in gel is obtained from the following formula (S2):

$\text{V}_{\text{r}}\text{=}\frac{{\text{m}_{\text{2}}}/{\text{ρ}_{\text{r}}}}{{\text{m}_{\text{2}}}/{\text{ρ}_{\text{r}}}\text{+}{\text{(}\text{m}_{\text{1}}\text{-}\text{m}_{\text{0}}\text{)}}/{\text{ρ}_{\text{s}}}}$ (S2)

where ρr and ρs represents the density of ENR (0.96 g cm^-3^) and the density of toluene (0.865 g cm^-3^), respectively.

The swelling ratio (W) can be obtained by the following equation (S3):

$\text{W=}\frac{\text{m}_{\text{1}}\text{-}\text{m}_{\text{0}}}{\text{m}_{\text{1}}}\text{×100\%}$ (S3)

**S1.3 Self-healing Performance Test**

For self-healing experiment, the dumbbellshaped sample (4 mm × 75 mm × 1 mm) was cut into two segements from its middle section by a clean knife, and then the incisions were immediately contacted together and healed at specific power of near-infrared (NIR) laser. The cutting gap of samples was observed on a polarizingmicroscope (Leica, Germany) during healing process. The tensile properties of healed samples were tested, and three specimens were measured for each sample. The healing efficiency (η) was calculated according to the formula (S4):

$\text{η=}\frac{\text{ε}_{\text{healed}}}{\text{ε}_{\text{original}}}\text{×100}$ (S4)

Among them, the ε_healed_ and ε_original_ represent the tensile strength of the original and healed samples, respectively.

**S1.4 Photothermal Performance Test**

For photothermal experiment, the thermal imaging camera (H16, HIKMICRO, China) was used to record the temperature of the sample surface at different NIR laser power settings. The efficiency of photothermal conversion was evaluated by the (average temperature)-time curve of the cuvette. The samples were placed in cuvette, irradiating with an NIR laser (808 nm, Lasever Inc., China) and recording the (average temperature)-time curve of the cuvette by thermal imaging camera. The process of the calculation are as follows (S5) [S2]:

$\sum_{\text{ⅈ}} \text{m}_{\text{i}}\text{C}_{\text{pi}}\frac{\text{dT}}{\text{dt}}\text{=}\text{Q}_{\text{s}}\text{+}\text{Q}_{\text{0}}\text{-}\text{Q}_{\text{loss}}$ (S5)

Mass and heat capacity of various components in the photothermal test system [cuvette: 4.88 g for m_1_ and 0.879 J g^-1^ K^-1^ for C_p1_; deionized water: ~2.52 g for m_2_ and 4.18 J g^-1^ K^-1^ for C_p2_; ECLM film: ~0.31g for m^3^ and 1.71J g^-1^ K^-1^ for Cp3]. Q_s_, Q_0_ and Q_loss_ represents the photothermal heat energy input by irradiating NIR laser to samples, the photothermal heat energy (≈ 0) input by irradiating NIR laser to the cuvette and the deionized water, and thermal energy lost to the surroundings, respectively. When the system temperature reaches maximum (Tmax), the system is regarded as being in balance (S6):

$\text{Q}_{\text{s}}\text{=}\text{Q}_{\text{loss}}\text{=hS∆}\text{T}_{\text{max}}\text{=hS(}\text{T}_{\text{max}}\text{-}\text{T}_{\text{surr}}\text{)}$ (S6)

Where h, Tsurr and S represents heat transfer coefficient, the temperature of the surroundings and the surface area of the container, respectively. The photothermal efficiency (η_pt_) can be calculated according to the following equation (S7):

$\text{η}_{\text{pt}}\text{=}\frac{\text{hS∆}\text{T}_{\text{max}}}{\text{Iδ(1-}\text{10}^{\text{-}\text{A}_{\text{808}}}\text{)}}$ (S7)

Where I, A_808_ and δ is incident laser power, the absorbance of the ECLE films at the wavelength of 808 nm, and the light transmittance (90%) of cuvette at the wavelength of 808 nm, respectively. To obtain the hS, a dimensionless driving force temperature, θ is introduced as the following (S8):

$\text{θ=}\frac{\text{T-}\text{T}_{\text{surr}}}{\text{T}_{\text{max}}\text{-}\text{T}_{\text{surr}}}$ (S8)

Where T is the system temperature. The sample system time constant (S9):

$\text{τ}_{\text{s}}\text{=}\frac{\text{Σ}_{\text{i}}\text{m}_{\text{i}}\text{C}_{\text{pi}}}{\text{hS}}$ (S9)

Therefore (S10):

$\frac{\text{dθ}}{\text{dt}}\text{=}\frac{\text{1}}{\text{τ}_{\text{s}}}\frac{\text{Q}_{\text{s}}}{\text{hS∆}\text{T}_{\text{max}}}\text{-}\frac{\text{θ}}{\text{τ}_{\text{s}}}$ (S10)

When the laser is turbed off, Qs = 0, thus (S11-S12):

$\frac{\text{dθ}}{\text{dt}}\text{=-}\frac{\text{θ}}{\text{τ}_{\text{s}}}$ (S11)

$\text{t=-}\text{τ}_{\text{s}}\text{lnθ}$ (S12)

The τs could be calculated from the slope of cooling time (t)-ln$\text{θ}$ curve. The total photothermal efficiency (η*), defined as the ratio of the heat produced by photothermal materials to the energy input of the laser, and its calculation formula is as follows (S13):

$\text{η}^{\text{*}}\text{=}\text{η}_{\text{pt}}\text{(1-}\text{10}^{\text{-A}_{\text{808}}}\text{)}$ (S13)

**S1.5 Photo-thermoelectric Performance Test**

Photo-thermoelectric test: A photo-thermoelectric generator (PTEG) system, consisting of a commercial Seeback thermoelectric generator (SP1848-27145), a heat sink, and ECLE-1, was utilized to demonstrate photo-thermoelectric application. PTEG system generated electricity at different NIR laser power settings. The temperature and output voltage of the PTEG system was recorded by the thermal imaging camera (H16, HIKMICRO, China) and the digital multimeter (Keithley DMM 7510, USA), respectively.

**S1.6 Conductivity Performance Test**

The conductivity testing was performed using an electrochemical workstation (DH7000C, DONGHUA, China). A bias voltage of 10 mV was applied, and the frequency range was set from 10^-1^ Hz to 10^5^ Hz. The samples (1×1 cm) were sandwiched between two stainless steel electrodes for the test. The calculation formula of conductivity (σ) is as follows (S14) [S3]:

$\text{ σ=}\frac{\text{L}}{\text{RS}}$ (S14)

where R, L, and S are the resistance, length, and cross-sectional area of the sample, respectively.

**S1.7 Ion Transference Number Test**

The ion transference number (T_Li_^+^) of the ECLE was measured using the AC impedance spectroscopy and the DC polarization. The polarization experiments was tested by electrochemical workstation with polarization voltage of 10 mV. The calculation formula of T_Li_^+^ is as follows (S15) [S4]

$\text{T}_{\text{Li+}}\text{=}\frac{\text{I}_{\text{s}}\text{(ΔV-}\text{I}_{\text{0}}\text{R}_{\text{0}}\text{)}}{\text{I}_{\text{0}}\text{(ΔV-}\text{I}_{\text{s}}\text{R}_{\text{s}}\text{)}}$ (S15)

Where I_0_ and R_0_ represent the initial currents and interfacial resistances, I_s_ and R_s_ represent steady currents and interfacial resistances. ΔV is the applied polarization voltage.

The activation energy (Ea) for ion transfer of ECLM-1 was calculated by Vogel-Tammann-Fulcher (VTF) equation (S16) [S5].

$\text{σ}\text{T}^{\text{1/2}}\text{=A exp}\left[ \frac{\text{-E}_{\text{a}}}{\text{R}\left( \text{T-}\text{T}_{\text{0}} \right)} \right]$ (S16)

Here, A and R were the pre-exponential factors. T_0_ was the reference temperature, which normally falls 30 K below the glass transition temperature (T_g_).

**S1.8 Water Evaporation Rate Test**

Water evaporation rate test: A container was filled with 100 mL 0.1 mol L^-1^ NaCl solution. The prepared ECLE (3×3 cm) was placed in a container surface to test the evaporation effect. An infrared lamp, as the light source, was placed above the ECLE. The weight and temperature change were recored by a balance and an infrared thermal camera in the evaporation process, respectively. the water evaporation rate ($\text{ν}$) was calculated using the following equations (S17):

$\text{ν=}\frac{\text{m}_{\text{1}}\text{-}\text{m}_{\text{0}}}{\text{St}}$ (S17)

Where, m_1_ and m_0_ is the original weight of the solution and the weight of the solution after evaporation, respectively. S and t is the heated area of the evaporator and the treated time of the evaporator, respectively.

**S1.9 Simulated Seawater Power Generation Test**

Simulated seawater power generation test: The ECLE was first cut into a rectangle shape (1×5 cm), and then the film was bent to a U-shape and bound on the polystyrene foam. The entire device was floated on 0.1 mol L^-1^ NaCl solution, with half of the film submerged in the solution. The real-time open-circuit voltage was recorded by the digital multimeter (Keithley DMM 7510).

**S1.10 Binding Energy Computational**

Binding energy computational methods: The structural optimizations were carried out using the DMol3 module on the Materials Studio software. The DFT Semi-core Pseudopots (DSPP) was employed to treat the core electrons, and the exchange-correlation effects were treated using the functional of Perdew-Burke-Ernzerhof (PBE) under the generalized gradient approximation (GGA) [S6, S7].

**S2 Supplementary Figures**

**
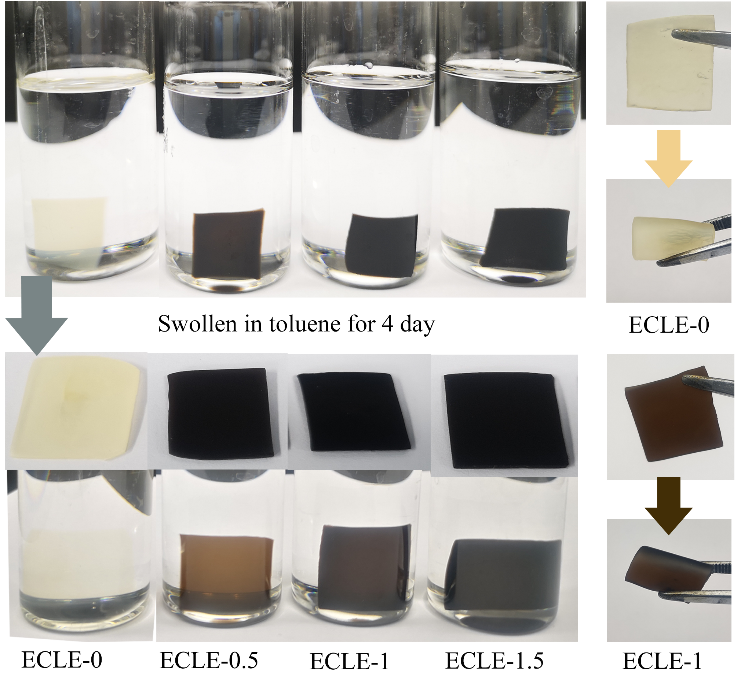
**

**Fig. S1** Comparison chart of before and after in equilibrium swelling experiment

**
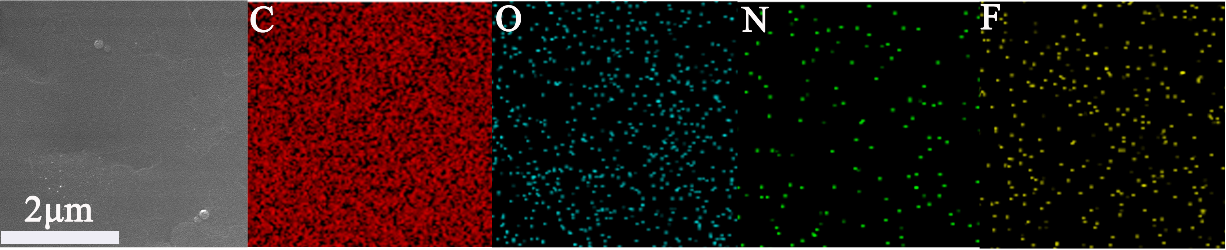
**

**Fig. S2** EDS of ECLE-1

**
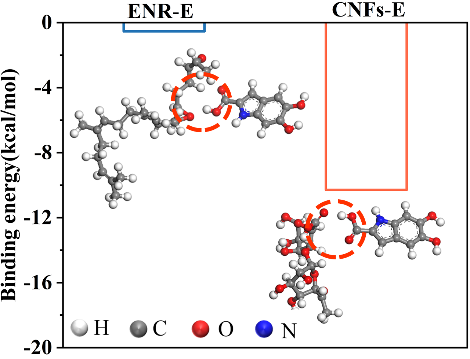
**

**Fig. S3** Binding energy of ENR-E and CNFs-E in ECLE systems, respectively

As depicts in Fig. S4a, when the strain exceeded 600%, the crystal diffraction peaks began to appear in the XRD curve, which showed the crystal diffraction peaks at 2θ = 13.9°, 17.4° and 20.2°, corresponding to the (200), (201) and (120) lattice planes of ENR crystallites, respectively [S8]. The crystallinity (Xc) of ECLE-1 was also calculated by equation (S18) [S9]:

$\text{X}_{\text{c}}\text{=}\frac{\text{A}_{\text{c}}}{\text{A}_{\text{c}}\text{+}\text{A}_{\text{a}}}$ (S18)

where Ac and Aa are the areas of crystalline and amorphous regions, respectively. The calculated result is shown in Fig S4b-h. The crystallinity of ECLE-1 increased from 2.98 of 600% to 23.61 of 1000%. The above results demonstrate that ECLE exhibits excellent SIC behavior. This may be due to the introduction of eumelanin, which increases the physical cross-linking points of the structural network and strengthens the interaction between ENR molecular chains, making it easier for the molecular chains to orient along the stretching direction and form crystalline domains during the stretching process.


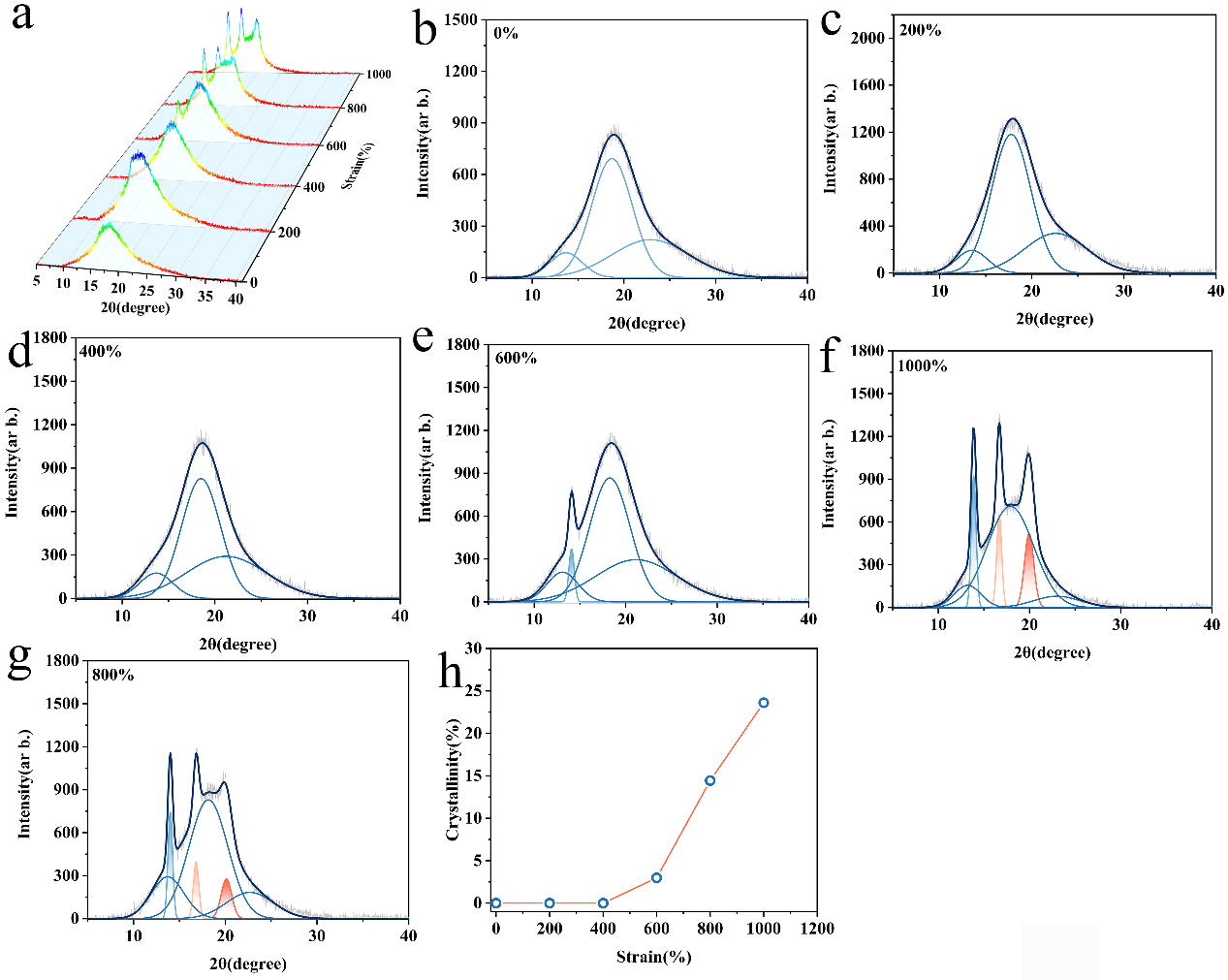


**Fig. S4** **a** XRD curves of ECLE-1 at different strain. **b-g** Gaussian fitting data of XRD curve at different strains of ECLE-1. **h** Crystallinity of ECLE-1

**

**

**Fig. S5** The stress, strain, toughness and Young’s modulus of ECLE

**

**

**Fig. S6** UV–Vis–NIR light transmission spectra of ECLE


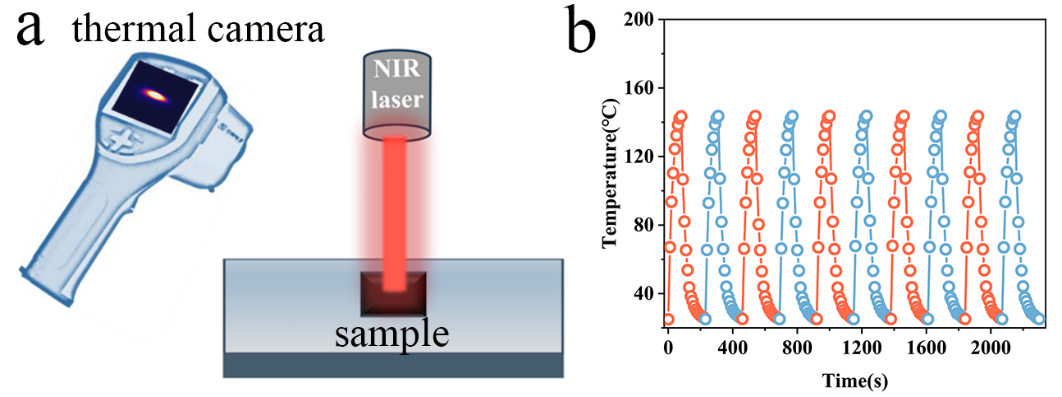


**Fig. S7** **a** Schematic measurement setup of photothermal conversion performance for ECLE. **b** Cyclic photothermal conversion performance of ECLE-1 under 0.62 W cm^-2^


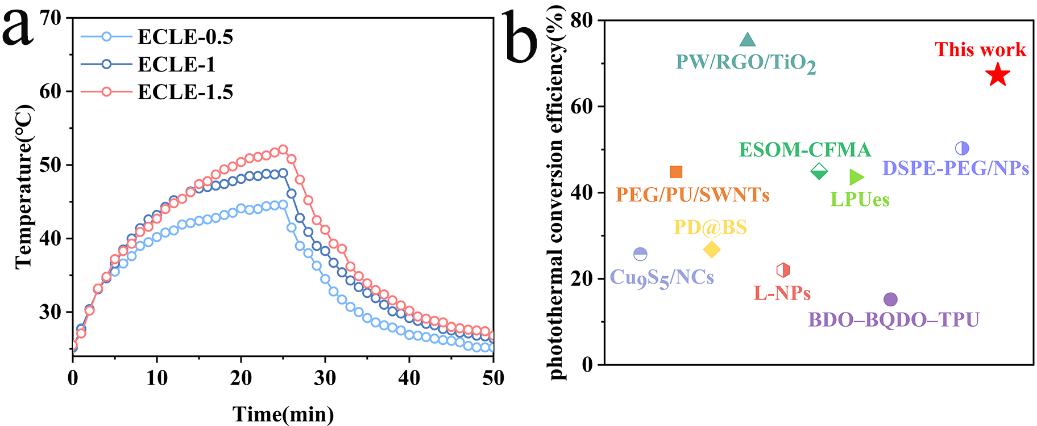


**Fig. S8** **a** The (average temperature)-time curve of the photothermal conversion efficiency of test cuvette containing ECLE under near-infrared laser irradiation and natural cooling conditions. **b** The photothermal conversion efficiency of ECLE were compared with other photothermal materials [S10-S18]

**
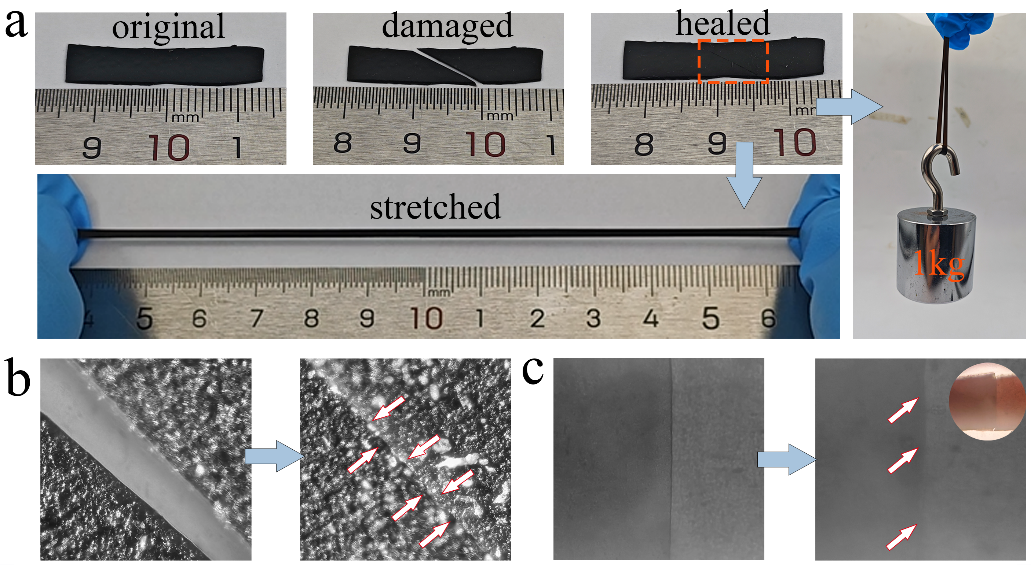
**

**Fig. S9** **a** Photographs of self-healing behaviors of ECLE-1 Optical microscope images of cut lines healed at 0.26 W/cm^2^ for 2 h. **b** surface. **c** internal

**
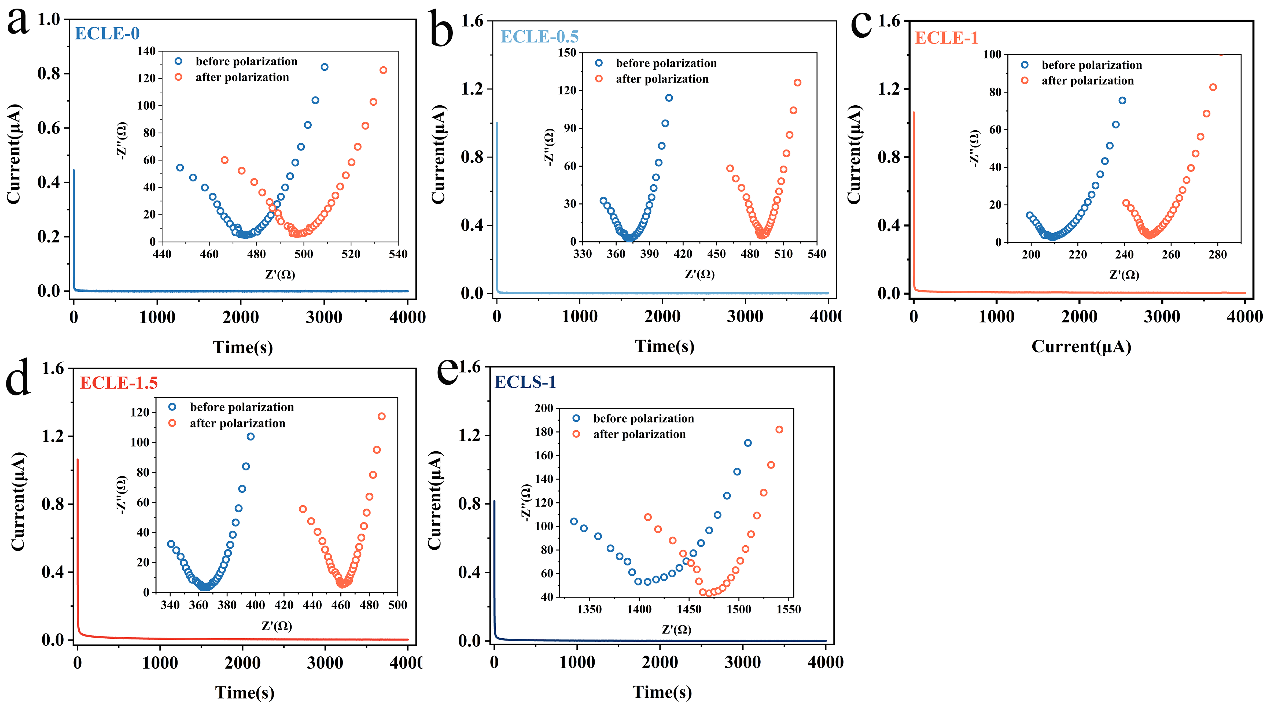
**

**Fig. S10** Current evolution under a polarization voltage of 10 mV. The inset shows the impedance spectra of the samples with before and after polarization: **a** ECLE-0; **b** ECLE-0.5; **c** ECLE-1; **d** ECLE-1.5; **e** ECLS-1





**Fig. S11** Arrhenius plot of the ionic conductivity of ECLE-1


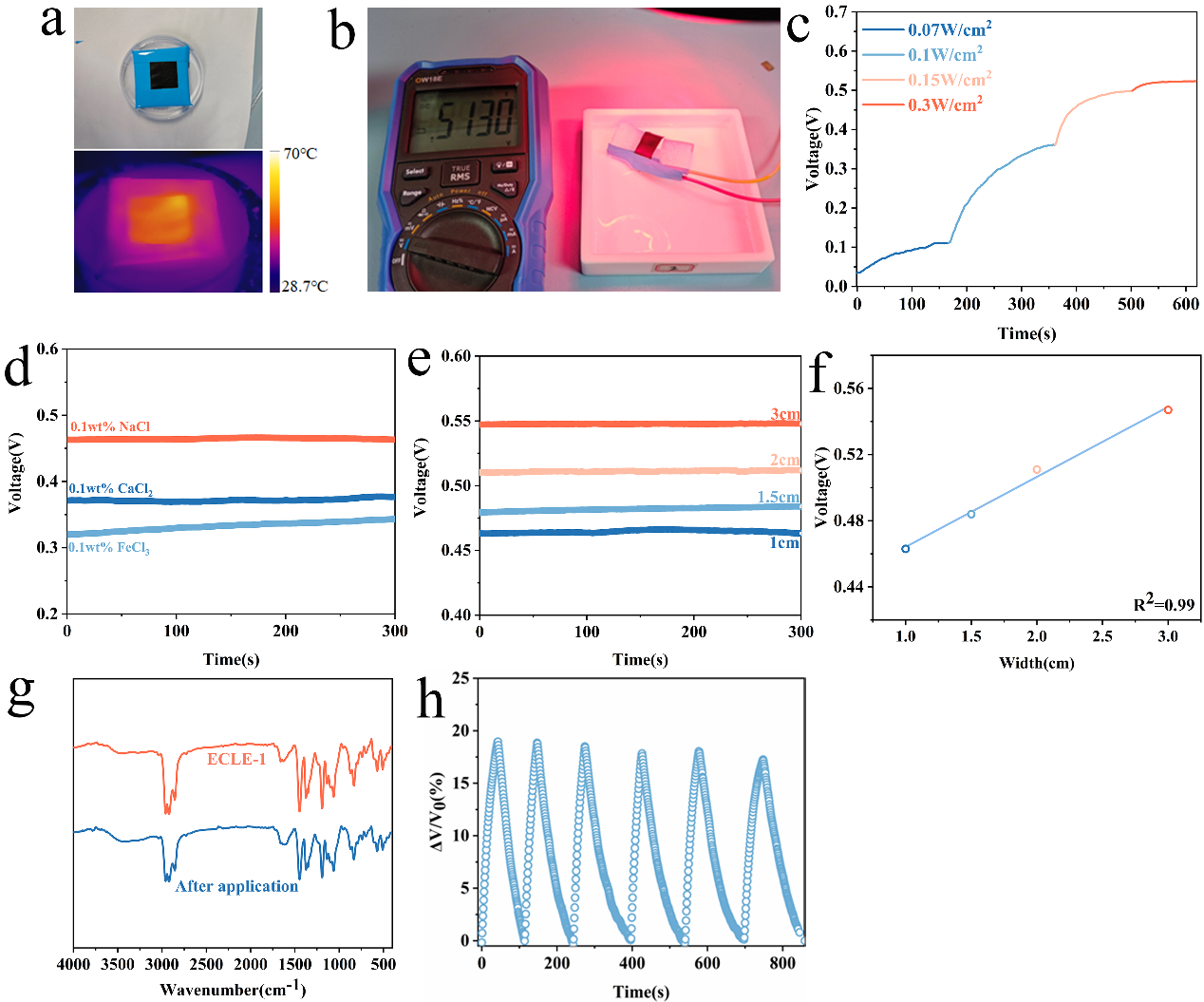


**Fig. S12** **a** The infrared image shows the temperature distribution on the film surface after illumination for 100 min. **b** Photograph of the ECLE-1 under 0.15 W/cm^2^ laser irradiation. **c** The output voltage of the ECLE-1 at different laser power. **d** The output voltage of the ECLE-1 at different cation. **e** The output voltage of the ECLE-1 at different width. **f** The output voltage of ECLE-1 versus width. **g** Comparison of infrared testing of ECLE-1 before and after 7 days of continuous output voltage testing. **h** The output voltage cyclic response of ECLE-1 to on-off of light





**Fig. S13** The output voltage of ECLE-1 reach 0.36V at river water under 0.15 W cm^-2^ NIR laser irradiation

The ECLE-1 was cut into two pieces, and then the two separate parts were made to fit together and immersed in 0.1 wt% NaCl solution (Fig. S13). After self-healing under an irradiation power of 0.26 W cm^-2^ for 2 h, the sample could be stretched to 700 % without fracturing.


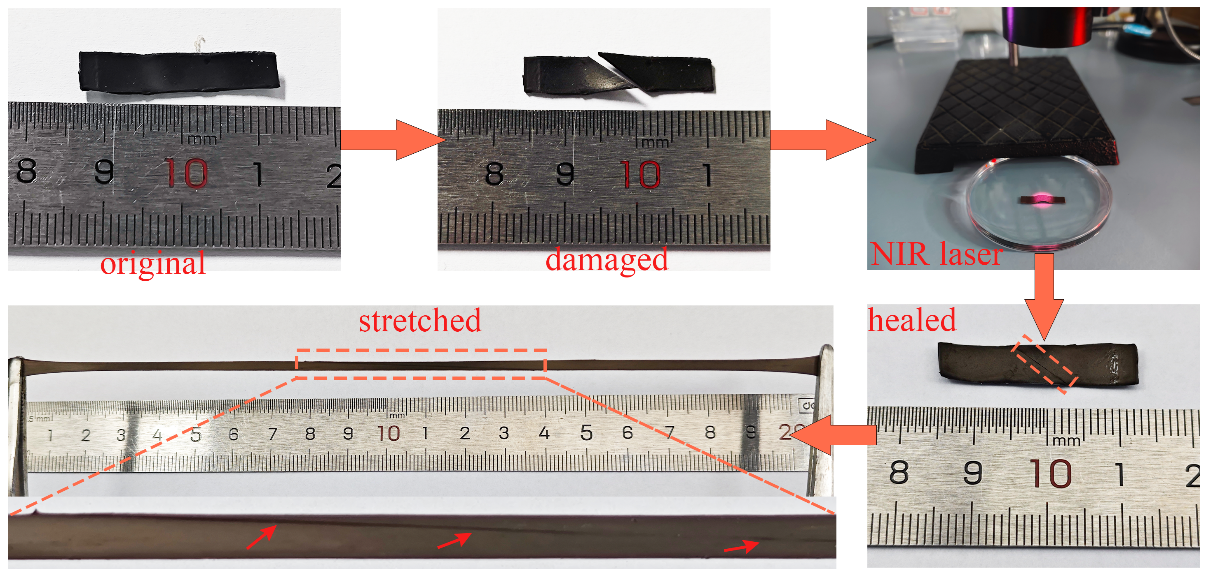


**Fig. S14** The self-healing process of ECLE-1 in a 0.1 wt% NaCl solution under an irradiation power of 0.26 W/cm² for 2 h


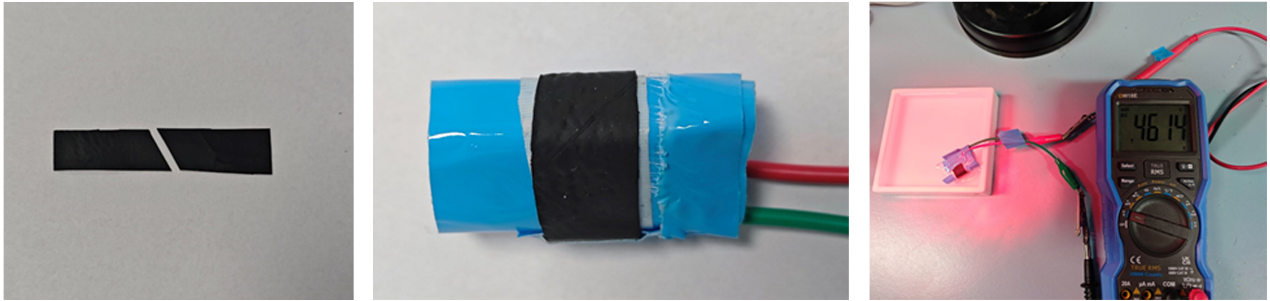


**Fig. S15** Photograph of the output voltage and self-healing process of healed ECLE-1 under an irradiation power of 0.26 W/cm² for 2 h





**Fig. S16** The output voltage of healed ECLE-1 under PH=8 conditions


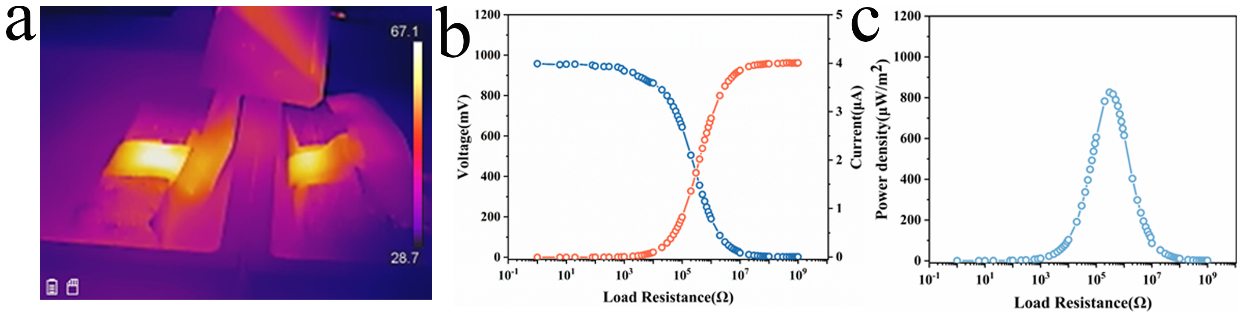


**Fig. S17** **a** Infrared images of the two ECLE-1 devices under an irradiation power of 0.15 W/cm². **b** Output current and voltage when the external resistance changes. **c** The calculation of power output under different resistance loads

**
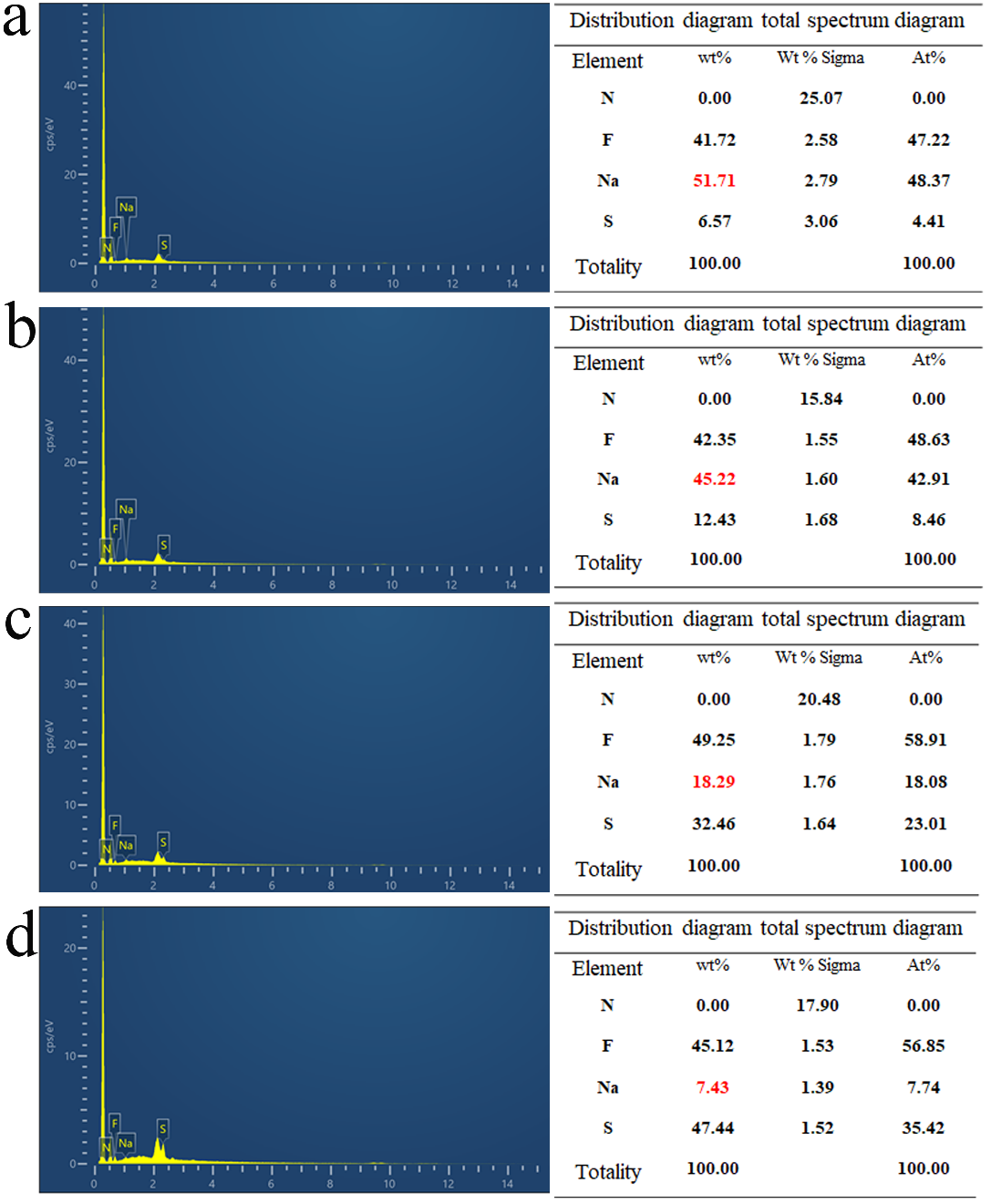
**

**Fig. S18** The distribution total spectrum diagram of EDS in different parts of ECLE-1: **a** 1; **b** 2; **c** 3; **d** 4

**
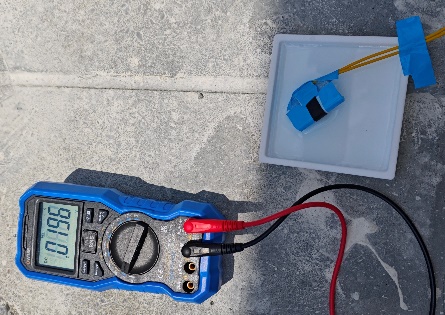
**

**Fig. S19** Photographs showing the output voltage of ECLE-1 without sun





**Fig. S20** Repeated charging and discharging of two ECLE-1 devices under sunlight

Supplementary References

1. L. Kong, Y. Yang, Z. Lin, B. Huang, L. Liao et al., A ENR-based conductive film integrating electricity-triggered self-healing, damage detection and high sensitivity for flexible sensors. Chem. Eng. J. **479**, 147624 (2024). <https://doi.org/10.1016/j.cej.2023.147624>
2. L. Li, Z. Lin, Z. He, Z. Su, L. Fu et al., Robust and flexible rubber composite with high photothermal properties achieved by in situ zdma assisted dispersion of eumelanin and its hydrophobic photothermal application. Small **3**, 2403553 (2024). <https://doi.org/10.1002/smll.202403553>
3. P. Zhou, W. Zhan, S. Shen, H. Zhang, Z. Zou et al., Strain‐stiffening, robust yet compliant ionic elastomer from highly entangled polymer networks and metal-oxygen interactions. Adv. Funct. Mater. **34**, 2402952 (2024). <https://doi.org/10.1002/adfm.202402952>
4. Z. Li, H. Huang, J. Zhu, J. Wu, H. Yang et al., Ionic conduction in composite polymer electrolytes: Case of peo:Ga-llzo composites. ACS Appl. Mater. Interfaces **11**, 784-791 (2018). <https://doi.org/10.1021/acsami.8b17279>
5. Y. Tan, H. Chen, W. Kang, X. Wang, Versatile light-mediated synthesis of dry ion-conducting dynamic bottlebrush networks with high elasticity, interfacial adhesiveness, and flame retardancy. Macromolecules **55**, 9715-9725 (2022). <https://doi.org/10.1021/acs.macromol.2c01234>
6. C. Wang, W. Li, D. Li, X. Zhao, Y. Li et al., High-performance solid-state lithium metal batteries of garnet/polymer composite thin-film electrolyte with domain-limited ion transport pathways. ACS Nano **18**, 32175-32185 (2024). <https://doi.org/10.1021/acsnano.4c11205>
7. Z. Sun, C. Dang, H. Zhang, Y. Feng, M. Jiang et al., Lignin powered versatile bioelastomer: A universal medium for smart photothermal conversion. Adv. Funct. Mater. **34**, 2405130 (2024). <https://doi.org/10.1002/adfm.202405130>
8. L. Imbernon, R. Pauchet, M. Pire, P. Albouy, S. Tencé-Girault et al., Strain-induced crystallization in sustainably crosslinked epoxidized natural rubber. Polymer **93**, 189-197 (2016). <https://doi.org/10.1016/j.polymer.2016.04.023>
9. X. Chen, L. Meng, W. Zhang, K. Ye, C. Xie et al., Frustrating strain-induced crystallization of natural rubber with biaxial stretch. ACS Appl. Mater. Interfaces **11**, 47535-47544 (2019). <https://doi.org/10.1021/acsami.9b15865>
10. H. Li, L. Zou, Q. Li, P. Chen, X. Quan et al., Vertically π-extended strong acceptor unit boosting near-infrared photothermal conversion of conjugated polymers toward highly efficient solar-driven water evaporation. J. Mater. Chem. A **11**, 2933-2946 (2023). <https://doi.org/10.1039/d2ta07628d>
11. J. Wang, X. Lin, R. Wang, Y. Lu, L. Zhang, Self‐healing, photothermal‐responsive, and shape memory polyurethanes for enhanced mechanical properties of 3d/4d printed objects. Adv. Funct. Mater. **33**, 2211579 (2022). <https://doi.org/10.1002/adfm.202211579>
12. H. Wang, J. Huang, W. Liu, J. Huang, D. Yang et al., Tough and fast light-controlled healable lignin-containing polyurethane elastomers. Macromolecules **55**, 8629-8641 (2022). <https://doi.org/10.1021/acs.macromol.2c01401>
13. C. Lu, Y. Liu, C. Wang, Q. Yong, J. Wang et al., An integrated strategy to fabricate bio-based dual-cure and toughened epoxy thermosets with photothermal conversion property. Chem. Eng. J. **433**, 134582 (2022). <https://doi.org/10.1016/j.cej.2022.134582>
14. X. Zhao, C. Huang, D. Xiao, P. Wang, X. Luo et al., Melanin-inspired design: Preparing sustainable photothermal materials from lignin for energy generation. ACS Appl. Mater. Interfaces **13**, 7600-7607 (2021). <https://doi.org/10.1021/acsami.0c21256>
15. S. Xi, M. Wang, L. Wang, H. Xie, W. Yu, 3d reduced graphene oxide aerogel supported tio2-x for shape-stable phase change composites with high photothermal efficiency and thermal conductivity. Sol. Energy Mater. Sol. Cells **226**, 111068 (2021). <https://doi.org/10.1016/j.solmat.2021.111068>
16. C. Zhang, D. Li, P. Pei, W. Wang, B. Chen et al., Rod-based urchin-like hollow microspheres of bi2s3: Facile synthesis, photo-controlled drug release for photoacoustic imaging and chemo-photothermal therapy of tumor ablation. Biomater. **237**, 119835 (2020). <https://doi.org/10.1016/j.biomaterials.2020.119835>
17. X. Du, J. Xu, S. Deng, Z. Du, X. Cheng et al., Amino-functionalized single-walled carbon nanotubes-integrated polyurethane phase change composites with superior photothermal conversion efficiency and thermal conductivity. ACS Sustainable Chem. Eng. **7**, 17682-17690 (2019). <https://doi.org/10.1021/acssuschemeng.9b03853>
18. Q. Tian, F. Jiang, R. Zou, Q. Liu, Z. Chen et al., Hydrophilic Cu_9_S_5_ Nanocrystals: A Photothermal Agent with a 25.7% Heat Conversion Efficiency for Photothermal Ablation of Cancer Cells in Vivo. ACS Nano **12**, 9761-9771 (2011). <https://doi.org/10.1021/nn203293t>
